# Research on a 3-DOF Motion Device Based on the Flexible Mechanism Driven by the Piezoelectric Actuators

**DOI:** 10.3390/mi9110578

**Published:** 2018-11-06

**Authors:** Bingrui Lv, Guilian Wang, Bin Li, Haibo Zhou, Yahui Hu

**Affiliations:** Tianjin Key Laboratory for Advanced Mechatronic System Design and Intelligent Control, Tianjin University of Technology, Tianjin 300384, China; 15122160890@163.com (B.L.); cnrobot@tjut.edu.cn (B.L.); zhouhaibo@tjut.edu.cn (H.Z.); huyahui@tjut.edu.cn (Y.H.)

**Keywords:** flexible mechanism, three degrees of freedom, matrix-based compliance modeling, piezoelectric actuator

## Abstract

This paper describes the innovative design of a three-dimensional (3D) motion device based on a flexible mechanism, which is used primarily to produce accurate and fast micro-displacement. For example, the rapid contact and separation of the tool and the workpiece are realized by the operation of the 3D motion device in the machining process. This paper mainly concerns the device performance. A theoretical model for the static performance of the device was established using the matrix-based compliance modeling (MCM) method, and the static characteristics of the device were numerically simulated by finite element analysis (FEA). The Lagrangian principle and the finite element analysis method for device dynamics are used for prediction to obtain the natural frequency of the device. Under no-load conditions, the dynamic response performance and linear motion performance of the three directions were tested and analyzed with different input signals, and three sets of vibration trajectories were obtained. Finally, the scratching experiment was carried out. The detection of the workpiece reveals a pronounced periodic texture on the surface, which verifies that the vibration device can generate an ideal 3D vibration trajectory.

## 1. Introduction

The flexible hinge motion platform has significant advantages such as high transmission efficiency, non-backlash, high resolution and high motion accuracy. It has important applications in electron microscopy, measurement and calibration, ultra-precision positioning platforms, micro force detection, micromanipulators, robots and other fields [1,2]. Therefore, a large number of micro–nano motion platforms based on flexible mechanisms have been designed in recent years [3,4,5,6]. Researchers are trying to use different strategies to design more practical vibration devices in certain fields. For example, ways to increase the size of the flexible hinge to increase the bandwidth of the device are used in designing nanopositioning modules for high-throughput nanomanufacturing applications [7]. The decoupled positioning platform with compound parallelogram flexures and a compound bridge-type displacement amplifier is more suitable for the field of microscopic operation [8]. The design of the 3-legged prismatic-prismatic-spherical (3PPS) parallel-kinematic configuration makes the working space of the high-payload flexible parallel robot reach a few millimeters and degrees, which has shown application value in the field of ultraviolet nanometer lithography [9]. The lever-based amplification is used to enhance the displacement of the mechanism of the parallel three degrees of freedom (3-DOF) positioning platform. The large displacement and high repeat positioning accuracy mechanism is more suitable for the micro–nano operation field [10]. Through the analysis of the influence of the quality and quantity of hinges on the performance of fast tool servo systems, it has been found that the single-hinge fast tool servo structure is more suitable for precision machining of the roll mold [11]. These areas require flexible devices with a wide range, high precision, high resolution, high reliability, and multiple degrees of freedom [12,13].

The application of a flexible mechanism in ultra-precision positioning platforms, electron microscopes and other precision instruments, requires that the motion device has a wide operational range [14,15]. There are several types of displacement amplification mechanisms: the lever type, the bridge type and the Scott-Russell mechanism. These basic amplifying mechanisms achieve superior performance through different combinations in practical applications. The self-guided displacement amplifying mechanism is obtained by combining a double composite guide rail and a bridge amplifying mechanism and can improve the output precision and range of the mechanism [1]. The double-rod amplifying structure based on the lever principle exhibits superior effects over the single-rod mechanism and improves the working range and natural frequency of the overall mechanism [16]. A mechanism consisting of multiple amplifiers has become a common approach. Lin and Zhang, respectively, designed a sub-millimeter working range of motion mechanisms. One motion mechanism is composed of multiple bridge amplifier structures [17], and the other motion mechanism is composed of a bridge amplifier and a lever compound amplifier [18]. The combination of two Scott–Russell and a half-bridge mechanism for double-stage displacement amplification provides a larger magnification ratio. The amplification ratios for the *x* and *y* directional motions can reach 5.2 and 5.4, respectively [19].

In addition, the flexible hinge mechanism requires the motion device to have high operational accuracy in applications such as micro gripper, micro-electro-mechanical system MEMS assembly, cell injection and other precision instruments. In general, most of high-precision positioning stages consist of two parts; actuators and guiding mechanisms. There are also some devices that use position feedback sensors to improve accuracy [20]. These devices can achieve an accuracy of approximately 30 nm using intuitive environmental force feedback during low-speed interactions [21]. The closed-loop positioning accuracy of the device can reach 600 nm, using a new type of repetitive compensation proportional integral differential (PID) controller combined with the inverted Prandtl–Ishlinskii model [22].

In order to increase processing efficiency, mechanisms are often required to have a higher natural frequency, based on the application of the flexible hinge mechanism, in the field of diamond turning and polishing. The 2-DOF motion device reduces mass through a compact design and its natural frequency reaches approximately 2900 Hz [23]. High-frequency motion devices also exhibit excellent performance in vibration-assisted turning [24]. Qu et al. designed a 2-DOF motion device. In order to have remote-center-of-motion (RCM) characteristics, the mass of the device is increased, and the natural frequency of the device is also reduced to 280.3 Hz [25]. The three-dimensional vibrating device for elliptical vibratory turning has a large motion range but its natural frequency is low [26]. When the motion device is designed, the input stiffness is usually determined due to the limitation of piezoelectric performance. Therefore, a simple structure and a small number of moving members are common choices for raising the natural frequency of the mechanism. This means that other performance may be degraded. For example, the addition of amplifiers and decoupling components increases the quality of the structure and causes the natural frequency to drop. In addition, as the input frequency increases, the output displacement of the general mechanism will also decay. These issues require a lot of research.

For some other indicators, such as decoupling, compact structure, high-stability and multiple-degrees-of-freedom, some scholars have undertaken deep research. Zhou et al. established a 2-DOF flexible mechanism, using the parallel kinematic constraint map method, to achieve totally geometric decoupling and actuator isolation [27]. Guo et al. realized the coupling compensation of the motion platform through the decoupling feedforward/feedback controller [28] and applied it to micro/nano positioning control [29]. Cai et al. improved the stability and plane motion capability of the mechanism through the design of the “T-shaped” flexible hinge mechanism [30]. Lee et al. used closed-loop feedback to deal with the decoupling problem, so that the operating bandwidth of the device reached 100 Hz [3]. Li et al. invented a compact series piezoelectric drive platform through a “Z-shaped” flexible hinge design [31]. In addition, Cai et al. designed a six-degree-of-freedom precision positioning of the motion platform [32]. Chen et al. designed a large-range compliant remote center of motion RCM stage with input/output decoupling [33].

In general, for motion platforms based on the flexible mechanism, a lot of research and exploration has been done on the range of motion, motion accuracy and natural frequency. Meanwhile, various motion platforms have been developed to suit different applications, but there are still some problems that remain unsolved. For example, because of the limitation of the spatial structure, the output stiffness problem of the output terminal has been given less consideration. In this case, some devices may become inefficient when a motion platform is used for precision machining. The output reliability, stability and operational accuracy of these devices are relatively decreased due to the complex cutting force. In addition, most of the existing devices only achieve open-loop control; however, the actual displacement detection and control at the output terminal are more difficult to accomplish. In this paper, a new type of 3-DOF motion device that can realize closed loop control with large output stiffness, and a self-guided characteristic of the output is designed. The main contents are as follows: first, the static and dynamic analysis of the device is carried out based on the analysis of the mechanism and characteristics of the device; second, the dynamic performance, axial linear motion performance and motion trajectory detection of the device are tested.

## 2. Overall Design of the System

### 2.1. Summary

**Figure 1:**
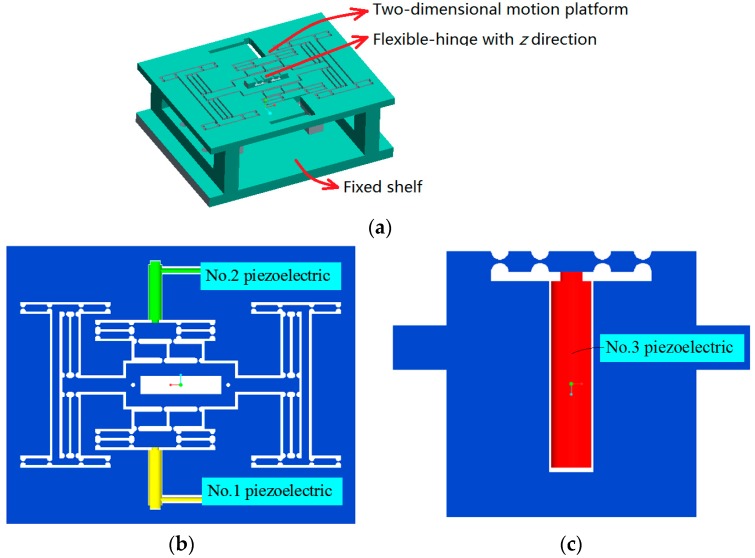
Schematic of a three degrees of freedom (3-DOF) motion device. (**a**) Overall design of the system; (**b**) two-dimensional (2D) motion platform (*x* and *y* directions); (**c**) flexible-hinge with *z* direction.

A 3-DOF motion device with *x y* and *z* three direction translations was designed in this study. As shown in Figure 1a, the 3-DOF motion device has the characteristics of a simple control, large output stiffness, and self-guided output. Meanwhile it also can realize closed loop control. The device is mainly a series connected by the two-dimensional (2D) motion platform including *x* and *y* directions, and the independent movement structure with *z* direction. The 2D motion platform is shown as Figure 1b, and the independent movement structure is shown as Figure 1c.

**Figure 2:**
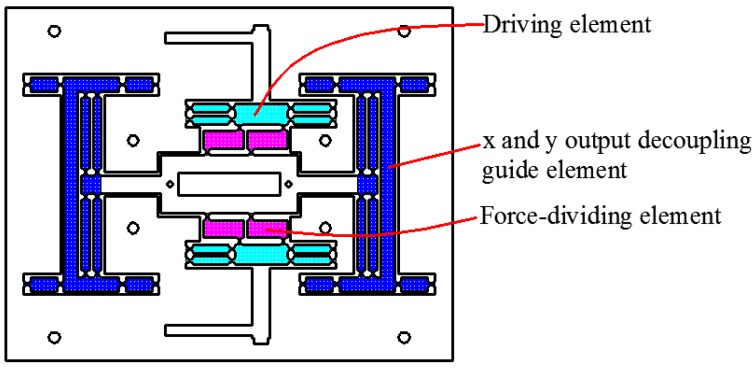
2D motion platform structure composition.

**Figure 3:**
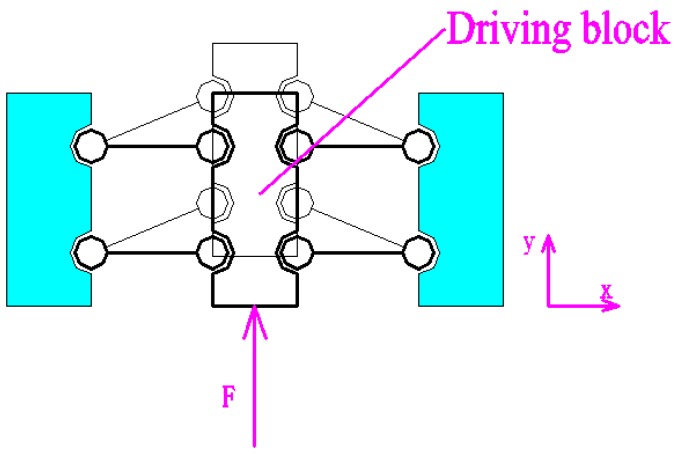
Principle of the driving element.

As shown in Figure 2, the 2D motion platform consists of a drive element, a force-removing element (consisting of four force parts), and x and y output decoupling guide elements. The driving element principle of the motion platform is shown in Figure 3. The principle of a double parallelogram is applied to ensure that the driving block moves steadily along the *y* axis under the action of the piezoelectric driving force, which can reduce the deflection error of the driving block caused by the piezoelectric installation error.

**Figure 4:**
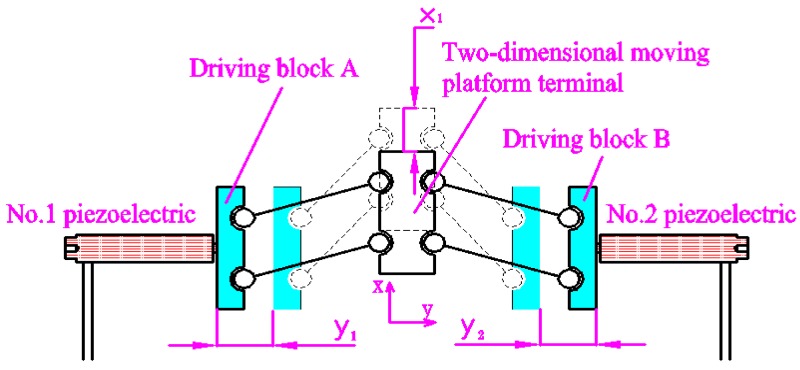
Principle of *x*-direction motion of 2D motion platform.

The force decomposition element of the three-dimensional (3D) motion device can separate the force of driving element into *x* and *y* directions so as to realize the *x* and *y* movement of the output platform by the different feeding strategies of the two driving blocks, block A and block B. The *x*-direction motion principle of a 2D motion platform is shown in Figure 4. When the No. 1 and No. 2 piezoelectric elements are extended or shortened at the same time at the same speed, the 2D motion platform produces displacement in the *x* direction. For example, block A and block B are respectively moved by distances *y*_1_ and *y*_2_. When *y*_1_ = *y*_2_, the output block of the 2D motion platform generates a displacement in the *x* direction. In fact, the equivalent rod of the force-dividing block is always inclined within the operating range of the device. When the piezoelectric element is extended, the 2D motion output terminal is always subjected to the component force along the *x*-positive direction, and it can only move upward along the *x*-direction. Owing to material rebound, when the piezoelectric element is shortened, the 2D output terminal moves down along the *x*-negative direction.

**Figure 5:**
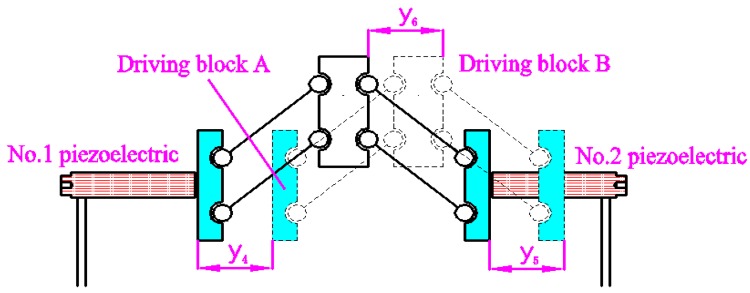
Principle of *y*-direction motion of 2D motion platform.

The *y*-direction motion principle of a 2D motion platform is shown in Figure 5. When the No. 1 piezoelectric element is extended or shortened, the No. 2 piezoelectric element is shortened or extended at the same speed at the same time. For example, the driving block A and block B move *y*_4_ and *y*_5_ to the right, respectively. When *y*_4_ = *y*_5_, the output block of the 2D motion platform generates a displacement in the *y*-direction.

### 2.2. x and y Output Decoupling Guide Element

**Figure 6:**
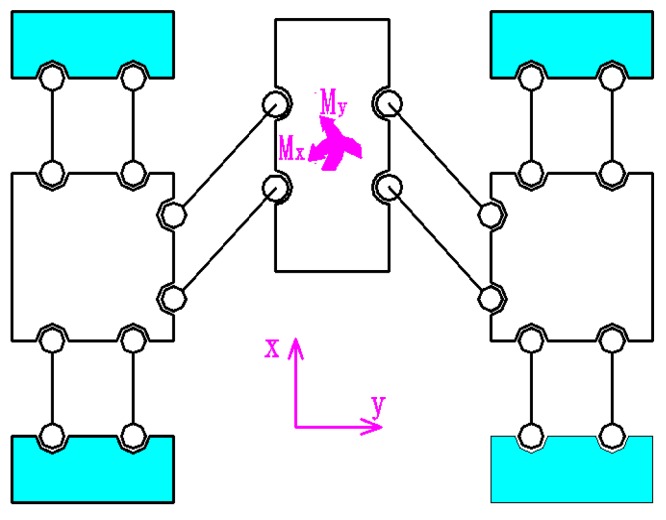
Common force conditions during work.

As shown in Figure 6, since the 2D motion platform is often subjected to the movements *M*_x_ and M_y_ during vibration cutting or polishing, this requires a large torsional stiffness at the output terminal of the 2D motion platform. In order to further enhance the device’s operational stability, reliability, output accuracy, and so that the actual displacement in the *x* and *y* directions can be detected at any time, a fully decoupled guidance structure for the 2D motion platform output terminal was designed.

**Figure 7:**
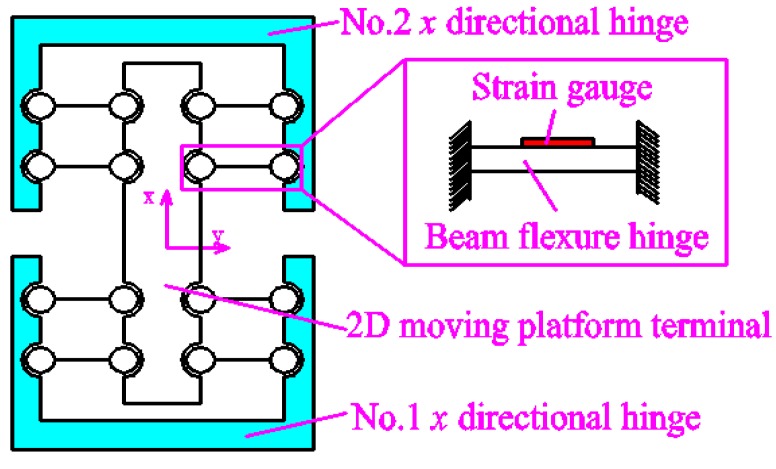
The x-guidance element principle.

The principle of the x-guidance element principle is shown in Figure 7. On the one hand, it can reduce the influence of force or moment on the output terminal and improve the output precision and output stiffness of the 2D motion platform, so as to improve the reliability of the device. On the other hand, detecting the displacement value of the x-guidance structure can reflect the actual displacement of the *x* direction of the 2D motion platform output terminal in real time or the strain gauge can be mounted on the beam flexible hinge to calculate its actual displacement in the *x* direction. Because the beam flexure hinge will reduce the rigidity of the mechanism, a circular flexure hinge is adopted in this paper.

**Figure 8:**
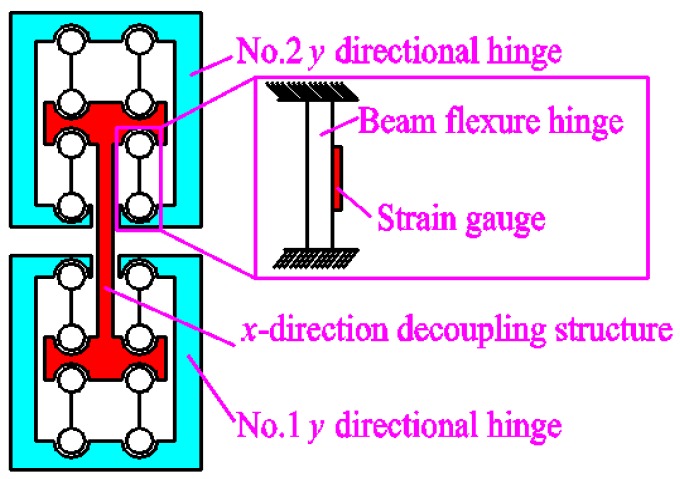
The *y*-guidance element principle.

The principle of the y-guidance structure is shown in Figure 8. Similar to the x-guidance structure function, it not only improves the *y*-direction output accuracy, but also improves output force conditions. In addition, it can realize the displacement detection of the 2D motion platform output terminal in the *y* direction. The detection method is similar to that of the *x*-direction. In order to improve the force performance of the output terminal as much as possible, this design adopts a straight round hinge. However, it should be explained that the power element mechanics midpoint of the device is not exactly the same as the mechanical midpoint of the x, y output decoupling guide element mechanics midpoint. This may result in slight deflection of the output block of the 2D mobile platform.

In this paper, the power part of the 3D motion device adopts the principle of serial-to-hybrid mixing, which has the advantages of both series and parallel structures. The parallel structure avoids the coupling of the piezoelectric actuator holder and the moving part, which further enhances the natural frequency of the device. In addition, the 2D motion platform adopts a parallel structure. The No. 1 and No. 2 piezoelectric actuators are arranged in a straight line, which is advantageous for the structure and the force symmetry. The decoupling guide structure adopts the principle of a parallelogram to ensure the parallel movement of the output terminal, which not only improves the output stiffness, but also improves the running accuracy of the device. The decoupling element characteristic decomposes the displacement of the output terminal into the *x* and *y* directions. The automatic displacement decomposition inside the device provides an aid for the detection and control of the output displacement of the device.

## 3. Analysis of Mechanism Characteristics

### 3.1. Static Analysis

#### 3.1.1. Calculation of Output Flexibility Using the MCM Method

(1)   Output Flexibility in the *x* and *y* Directions

**Figure 9:**
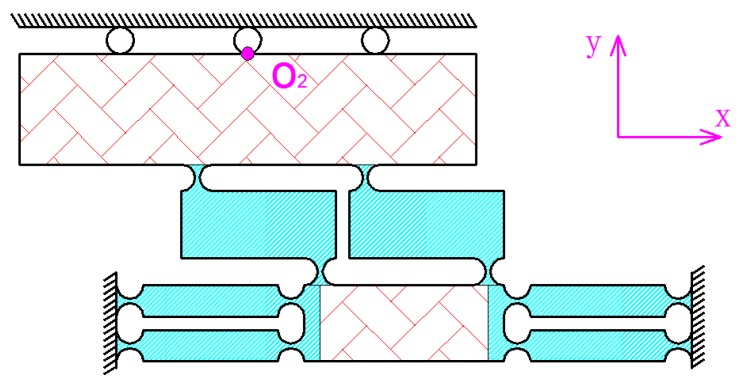
x and y power part.

The matrix-based compliance modeling (MCM) method is given in the Supplementary Materials [34]. The driving element and force-dividing element of the 2D platform provide impetus for the output platform, and the x and y output decoupling guide element improves the rigidity of the output platform. On the premise that the output center point of the power part is consistent with the mechanics center of the x and y output decoupling guide element, in order to calculate the whole output terminal flexibility, 2D motion platform is divided into two parts: the power part and the decoupling detection part. The power part of x and y is shown in Figure 9. In Figure 9, the red marked area has little influence on its static performance. Therefore, this part is considered to be rigid.

Some symbols are defined for convenient description.

The $C_{m}^{R}$ is the flexibility matrix, which corresponds to the flexibility of the circular flexure hinge with the output terminal point *m*. It can be obtained according to Supplementary Materials Equation (S5). In this paper, the possible values of *m* include: $A,C,A_{1},C_{1},A_{1}^{\prime }$, $C_{1}^{\prime }$, $A_{2},C_{2},A_{3},C_{3},A_{4},C_{4}$.

The $C_{n}^{L}$ is the flexibility matrix, which corresponds to the flexibility of the straight beam flexure hinge with the output terminal point *n*. It can be obtained according to Supplementary Materials Equation (S6). In this paper, the possible values of *n* include: $B,D,B_{1},B_{2},D_{1},B_{1}^{\prime }$, $B_{3},B_{4}$.

The $T_{N}^{V}$ is a position matrix generated by moving the coordinate system with *N* as the coordinate origin to the coordinate system with *V* as the origin. It can be obtained according to Supplementary Materials Equations (S7)–(S9). In this paper, the possible values of *V* include: $O_{K1},O_{K2},$ $O_{1},O_{3},O_{4}$. The possible values of *N* include: $A,B,C,D,A_{1},B_{1},C_{1},D_{1},A_{1}^{\prime },B_{1}^{\prime },C_{1}^{\prime },A_{2},B_{2},C_{2},A_{3},B_{3},C_{3},A_{4},$ $B_{4},C_{4},A_{4}^{\prime },B_{4}^{\prime },C_{4}^{\prime },O_{K1},O_{K2}$.

**Figure 10:**
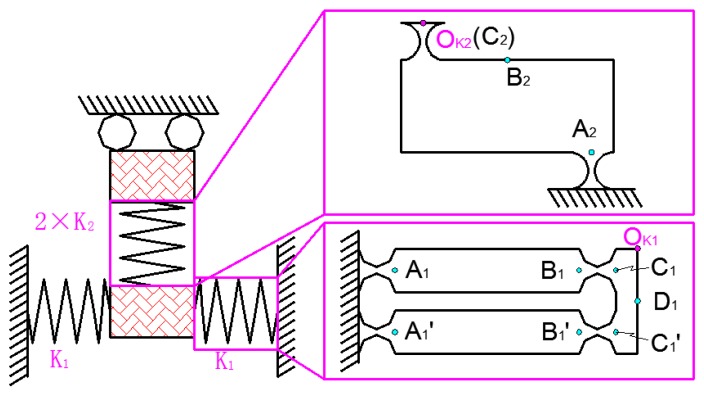
Static analysis of x and y power part.

For example, in Figure 10, the flexibility matrix $C_{A_{1}}^{R}$ of the straight circular hinge at point $A_{1}$ can be obtained by the Supplementary Materials Equation (S5). The position matrix $T_{A_{1}}^{O_{K1}}$ from point $A_{1}$ to point $O_{K1}$ can be obtained by the Supplementary Materials Equations (S7)–(S9). The flexibility of the straight circular hinge at the point $O_{K1}$ can be obtained by $T_{A_{1}}^{O_{K1}}*C_{A_{1}}^{R}*{\left(T_{A_{1}}^{O_{K1}}\right)}^{T}$.

In addition, if two hinges are connected in series, it can be expressed as $C_{m}=C_{m1}+C_{m2}$. If two hinges are connected in parallel, it can be expressed as $C_{m}={\left(C_{m1}^{-1}+C_{m2}^{-1}\right)}^{-1}$, where the flexibility of the two hinges is $C_{m1}$ and $C_{m2}$ respectively, $C_{m}$ is the total flexibility of the two flexible hinges.

A static analysis of the power portions of *x* and *y* is shown in Figure 10. The half-output flexibility of the power part can be simplified as a parallel connection of two flexible rods with stiffness *K*_1_ and then in series with the rod with stiffness 2 * *K*_2_. The $K_{1}$ model can be expressed as rod $A_{1}B_{1}C_{1}$ and rod $A_{1}^{\prime }B_{1}^{\prime }C_{1}^{\prime }$ in parallel and then in series with the rod $D_{1}$. Where the stiffness of rod $A_{1}B_{1}C_{1}$ can be expressed as $C_{A_{1}}$, $C_{B_{1}}$ and $C_{C_{1}}$ in series; the stiffness of rod $A_{1}^{\prime }B_{1}^{\prime }C_{1}^{\prime }$ can be expressed as $C_{A_{1}^{\prime }}$, $C_{B_{1}^{\prime }}$ and $C_{C_{1}^{\prime }}$ in series. The $K_{2}$ model can be expressed as $C_{A_{1}}$, $C_{B_{1}}$ and $C_{C_{1}}$ in series. Therefore, the flexibility matrix of *K*_1_ and *K*_2_ can be expressed as:

(1) $$\left\{ \begin{array}{l} C_{K_{1}}=({\left(T_{A_{1}}^{O_{K1}}*C_{A_{1}}^{R}*{\left(T_{A_{1}}^{O_{K1}}\right)}^{T}+T_{B_{1}}^{O_{K1}}*C_{B_{1}}^{L}*{\left(T_{B_{1}}^{O_{K1}}\right)}^{T}+T_{C_{1}}^{O_{K1}}*C_{C_{1}}^{R}*{\left(T_{C_{1}}^{O_{1}}\right)}^{T}\right)}^{-1}\\ +{\left(T_{A_{1}^{\prime }}^{O_{K1}}*C_{A_{1}^{\prime }}^{R}*{\left(T_{A_{1}^{\prime }}^{O_{K1}}\right)}^{T}+T_{B_{1}^{\prime }}^{O_{K1}}*C_{B_{1}^{\prime }}^{L}*{\left(T_{B_{1}^{\prime }}^{O_{K1}}\right)}^{T}+T_{C_{1}^{\prime }}^{O_{K1}}*C_{C_{1}^{\prime }}^{R}*{\left(T_{C_{1}^{\prime }}^{O_{1}}\right)}^{T}\right)}^{-1}{)}^{-1}+T_{D_{1}}^{O_{K1}}*C_{D_{1}}^{L}*{\left(T_{D_{1}}^{O_{1}}\right)}^{T}\\ C_{K_{2}}=T_{Z2}*(T_{A_{2}}^{O_{K_{2}}}*C_{A_{2}}^{R}*{\left(T_{A_{2}}^{O_{K_{2}}}\right)}^{T}+T_{B_{2}}^{O_{K_{2}}}*C_{B_{2}}^{L}*{\left(T_{B_{2}}^{O_{K_{2}}}\right)}^{T}+T_{C_{2}}^{O_{K_{2}}}*C_{C_{2}}^{R}*{\left(T_{C_{2}}^{O_{K_{2}}}\right)}^{T})*T_{Z2} \end{array}\right.$$

where $T_{Z_{2}}$ is the coordinate transformation matrix of 90° around *z*-axis.

The half-output flexibility of the power part of a 2D motion platform *C*_DH_ can be expressed as:

(2) $$C_{\mathrm{DH}}=T_{O_{K1}}^{O_{K2}}*(({\left(C_{K_{1}}\right)}^{-1}+{\left(T_{Y}*C_{K_{1}}*{\left(T_{Y}\right)}^{T}\right)}^{-1})*{\left(T_{O_{K1}}^{O_{K2}}\right)}^{T}+2*C_{K_{2}}$$

where *T_Y_* is the coordinate transformation matrix of 180° around *y*-axis. The overall flexibility of the power part of a 2D motion platform is symmetric about the *x*-axis. Therefore, *C*_D_ can be expressed as:

(3) $$C_{D}={\left((C_{\mathrm{DH}}{)}^{-1}+{\left(T_{X}*C_{\mathrm{DH}}*{\left(T_{X}\right)}^{T}\right)}^{-1}\right)}^{-1}$$

where *T_X_* is the coordinate transformation matrix of 180° around the *x*-axis.

**Figure 11:**
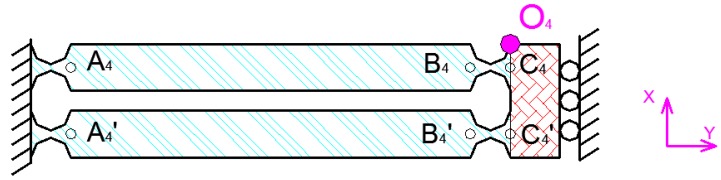
Analysis of *x*-direction hinge.

In order to simplify the calculation, when the *x*-direction statics analysis is performed, it can be considered that the *y*-direction hinge is a rigid body. When the deformation of the *y*-direction hinge is not considered in the *x*-direction, the *x*-direction flexibility of the x and y output decoupling guide element can be considered as two hinges connected in series as shown in Figure 11. Where the flexibility of one of the hinges can be expressed as:

(4) $$\begin{array}{l} C_{X}=({\left(C_{1X}\right)}^{-1}+{\left(T_{X}*C_{1X}*{\left(T_{X}\right)}^{T}\right)}^{-1})\\ C_{1X}=({\left(T_{A_{4}}^{O_{4}}*C_{A_{4}}^{R}*{\left(T_{A_{4}}^{O_{4}}\right)}^{T}+T_{B_{4}}^{O_{4}}*C_{B_{4}}^{L}*{\left(T_{B_{4}}^{O_{4}}\right)}^{T}+T_{C_{4}}^{O_{4}}*C_{C_{4}}^{R}*{\left(T_{C_{4}}^{O_{4}}\right)}^{T}\right)}^{-1}\\ +{\left(T_{A_{4}^{\prime }}^{O_{4}}*C_{A_{4}^{\prime }}^{R}*{\left(T_{A_{4}^{\prime }}^{O_{4}}\right)}^{T}+T_{B_{4}^{\prime }}^{O_{4}}*C_{B_{4}^{\prime }}^{L}*{\left(T_{B_{4}^{\prime }}^{O_{4}}\right)}^{T}+T_{C_{4}^{\prime }}^{O_{4}}*C_{C_{4}^{\prime }}^{R}*{\left(T_{C_{4}^{\prime }}^{O_{4}}\right)}^{T}\right)}^{-1}{)}^{-1} \end{array}$$

where *T_X_* is the coordinate transformation matrix of 180° around *x*-axis.

**Figure 12:**
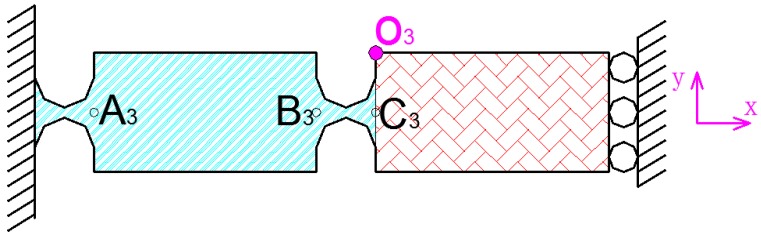
Analysis of *y*-direction hinge.

Similarly, when the *y*-direction statics analysis is performed, it can be considered that the *x*-direction hinge is a rigid body. When the deformation of the *x*-direction hinge is not considered in the *y*-direction, the *y*-direction flexibility of x and y output decoupling guide element can be considered as four hinges connected in series, as shown in Figure 12. The flexibility of one of the hinges can be expressed as:

(5) $$\begin{array}{l} C_{Y}=({\left(T_{A_{3}}^{O_{3}}*C_{A_{3}}^{R}*{\left(T_{A_{3}}^{O_{3}}\right)}^{T}+T_{B_{3}}^{O_{3}}*C_{B_{3}}^{R}*{\left(T_{B_{3}}^{O_{3}}\right)}^{T}+T_{C_{3}}^{O_{3}}*C_{C_{3}}^{R}*{\left(T_{C_{3}}^{O_{3}}\right)}^{T}\right)}^{-1}\\ +T_{Y}*{\left(T_{A_{3}}^{O_{3}}*C_{A_{3}}^{R}*{\left(T_{A_{3}}^{O_{3}}\right)}^{T}+T_{B_{3}}^{O_{3}}*C_{B_{3}}^{R}*{\left(T_{B_{3}}^{O_{3}}\right)}^{T}+T_{C_{3}}^{O_{3}}*C_{C_{3}}^{R}*{\left(T_{C_{3}}^{O_{3}}\right)}^{T}\right)}^{-1}*T_{Y}{)}^{-1} \end{array}$$

where *T_Y_* is the coordinate transformation matrix of 180° around the *y*-axis.

In summary, the output flexibility of the flexible hinge device in *x* and *y* directions can be expressed as:

(6) $$\left\{ \begin{array}{l} C_{\mathrm{WX}}=((C_{D}{)}^{-1}+(2*C_{X}{)}^{-1}{)}^{-1}\\ C_{\mathrm{WY}}={\left({\left(C_{D}\right)}^{-1}+{\left(4*C_{Y}\right)}^{-1}\right)}^{-1} \end{array}\right.$$

(2)   Output flexibility in the *z* direction

**Figure 13:**
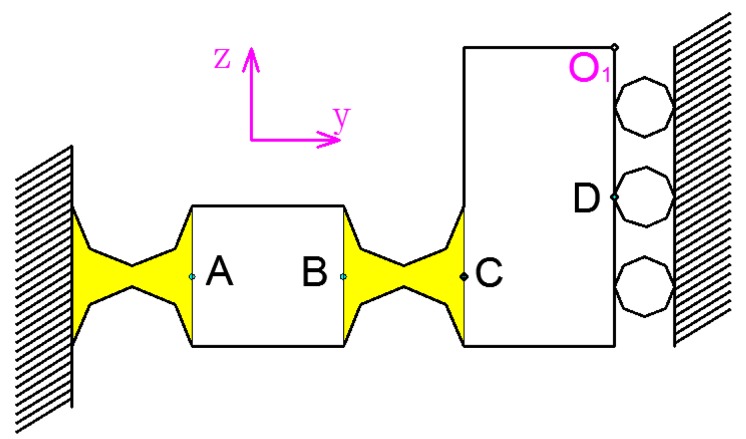
Analysis of *z*-direction hinge.

In order to simplify the calculation, when the *z*-direction statics analysis is performed, it can be considered that the *x* and *y* directions hinge is a rigid body. When the *z*-direction statics analysis is performed, the influence of 2D motion platforms flexibility in the *z* direction is not considered, so only the flexibility of the *z*-direction hinge is considered. Since the *z*-axis hinge is symmetric, only half of the structure needs to be studied. The *z*-direction hinge can be considered as a series connection of two circular flexure hinges and two beam flexure hinges. A static analysis of a half *z*-direction hinge is shown in Figure 13. The output flexibility of the *z*-direction hinge can be expressed as:

(7) $$\left\{ \begin{array}{l} C_{Z}={\left({\left(C_{O_{1}}\right)}^{-1}+{\left(T_{Z}*C_{O_{1}}*{\left(T_{Z}\right)}^{T}\right)}^{-1}\right)}^{-1}\\ C_{o_{1}}=T_{A}^{O_{1}}*C_{A}^{R}*{\left(T_{A}^{O_{1}}\right)}^{T}+T_{B}^{O_{1}}*C_{B}^{L}*{\left(T_{B}^{O_{1}}\right)}^{T}+T_{C}^{O_{1}}*C_{C}^{R}*{\left(T_{C}^{O_{1}}\right)}^{T}+T_{D}^{O_{1}}*C_{D}^{L}*{\left(T_{D}^{O_{1}}\right)}^{T} \end{array}\right.$$

where *T_z_* is the coordinate transformation matrix of 180° around *z*-axis.

When the flexibility of the *z*-direction hinge mechanism is calculated, the influence of 2D motion platform on the *z*-direction flexibility can be ignored. Therefore, the flexibility of the *z*-direction hinge device can be expressed as:

(8) $$C_{wz}=C_{z}$$

#### 3.1.2. Finite Element Analysis (FEA)

**Figure 14:**
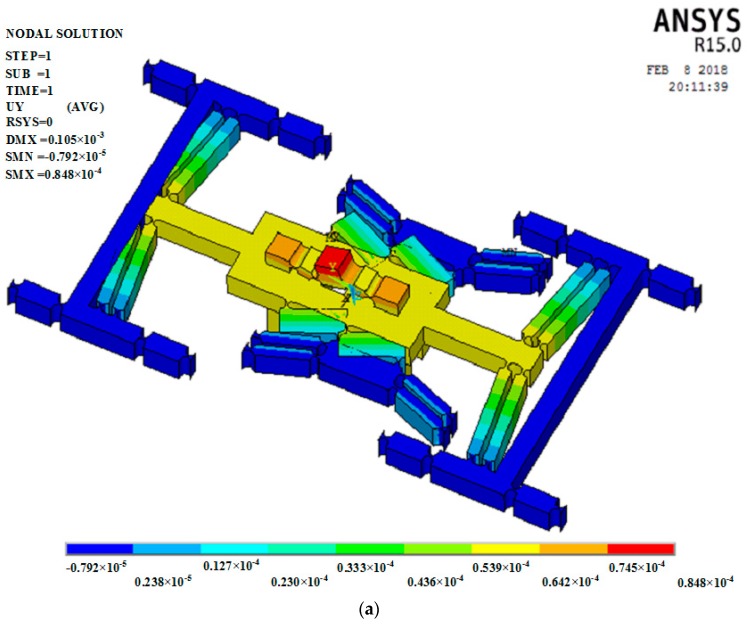

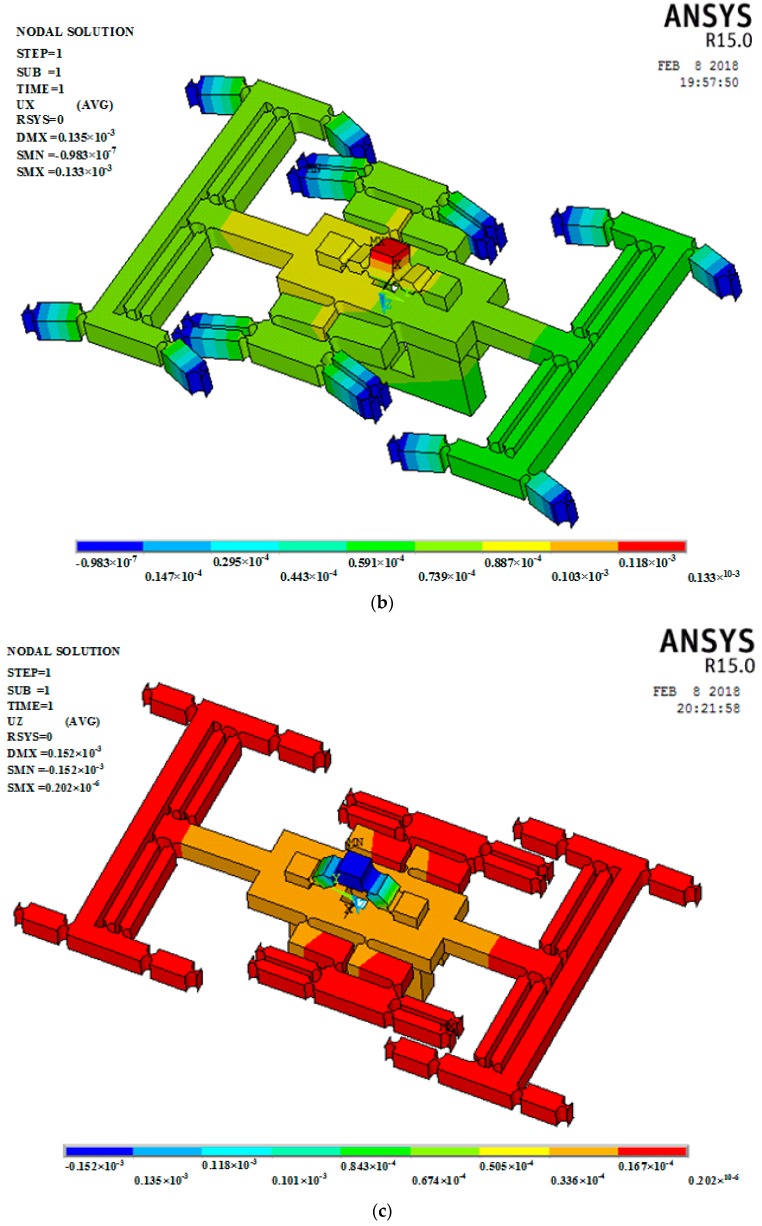
Finite element analysis of output compliance. (**a**) Displacement nephogram in *x* direction; (**b**) displacement nephogram in *y* direction; (**c**) displacement nephogram in *z* direction.

The structure parameters: *t* = 0.7 mm, *r* = 2 mm, *h* = 5 mm, *b* = 10 mm, *w* = 10 mm. As shown in Figure 14, full constraints are applied to all faces in the model that are attached to the fixture. Solid model 10 node 187 elements are used in the finite element model. The material is an aluminum alloy with elasticity, linearity and isotropy. The elastic modulus is 70 MPa. Poisson’s ratio is 0.3. A force of 100 N is applied in three directions, *x y* and *z,* respectively. The displacement nephograms for all directions are shown in Figure 14. The output compliance of the MCM method analysis results and FEA results are given in Table 1.

### 3.2. Dynamic Analysis

#### 3.2.1. Theoretical Model

**Figure 15:**
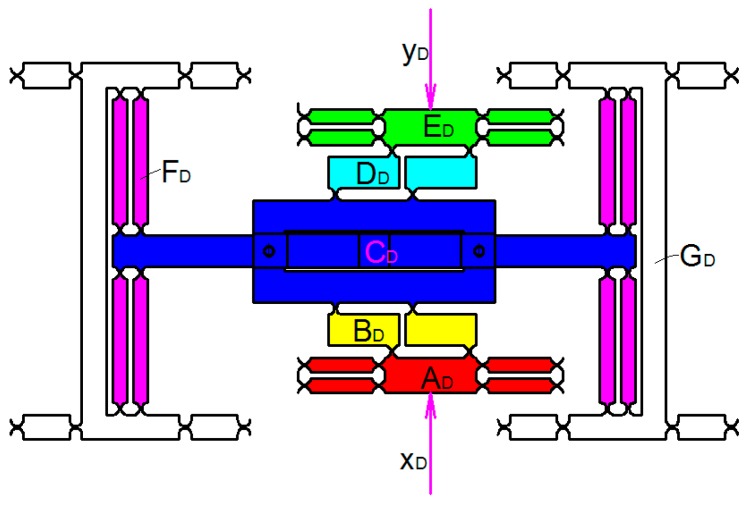
Three-dimensional motion device structure.

The FEA was performed prior to theoretical analysis, but is given later in this paper. According to the FEA, since the first and second natural frequencies of the 3-DOF motion device occur in the plane *xoy*, it is only necessary to analyze the natural frequency of device when the 2D motion platform has input signal. Using the energy method to establish the dynamic model of the flexible hinge mechanism, the natural frequency can be obtained according to the stiffness matrix *K* and quality matrix *M*. As shown in Figure 15, the 3D motion device can be divided into six units of $A_{D}$~$G_{D}$. Where $x_{D}$ and $y_{D}$ are the generalized displacements of the two drive motions respectively. Thus, the energy is generated in each unit of the three-dimensional motion device. The energy of each unit was calculated to obtain the total energy.

**Figure 16:**
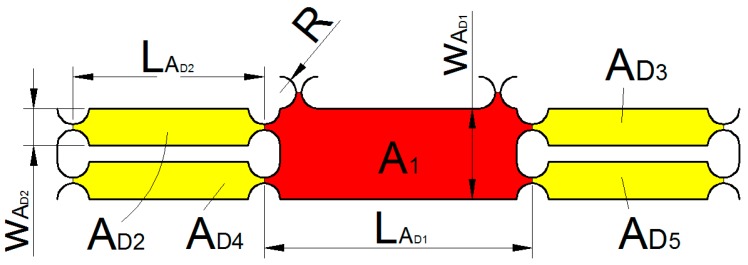
Unit $A_{D}$ structure.

As shown in Figure 16, unit $A_{D}$ can be divided into five parts of $A_{D1}$~$A_{D5}$. The kinetic energy of the unit $A_{D1}$ can be expressed as:

(9) $$T_{A_{D1}}=\frac{1}{2}m_{A_{D1}}{\dot{x_{D}}}^{2}$$

where $\dot{x_{D}}$ is the speed of the part $A_{D1}$, $m_{A_{D1}}$ is the mass of the part $A_{D1}$.

$m_{A_{D1}}$ can be expressed as:

(10) $$m_{A_{D1}}=(L_{A_{D1}}-2R)w_{A_{D1}}b\rho +3\left[2w_{A_{D1}}R-\pi R^{2}\right]b\rho$$

where ρ is the material density, b is the thickness of the 2D motion mechanism.

The kinetic energy of the unit $A_{D2}$ can be expressed as:

(11) $$T_{A_{D2}}=\frac{1}{2}I_{A_{D2}}{\left(\frac{\dot{x_{D}}}{L_{A_{D2}}}\right)}^{2}$$

where $I_{A_{D2}}$ is the rotational inertia of the part $A_{D2}$.

$I_{A_{D2}}$ can be expressed as:

(12) $$I_{A_{D2}}=m_{A_{D2}}\left[\frac{1}{3}{\left(L_{A_{D2}}-2R\right)}^{2}+(L_{A_{D2}}-2R\right)]R+R^{2}]$$

where $m_{A_{D2}}$ is the mass of the part $A_{D2}$.

$m_{A_{D2}}$ can be expressed as:

(13) $$m_{A_{D2}}=(L_{A_{D2}}w_{A_{D2}}-\pi R^{2})b\rho$$

**Figure 17:**
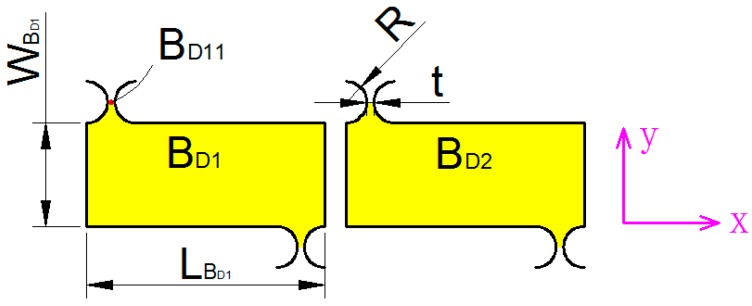
Unit $B_{D}$ structure.

As shown in Figure 17, the $B_{D11}$ point along *x*-axis and *y*-axis motion displacement of the local coordinate system can be expressed as:

(14) $$\{\begin{array}{c} x^{\prime }=\frac{x+y}{2}\\ y^{\prime }=\frac{x-y}{2} \end{array}$$

Since the motion of the unit $B_{D}$ is extremely complicated, this paper only considers the translational motion. Therefore, the kinetic energy of the unit $B_{D1}$ can be expressed as:

(15) $$T_{B_{D1}}=\frac{1}{2}m_{B_{D1}}\left[\lambda_{1}{\left(\frac{\dot{x_{D}}+\dot{y_{D}}}{2}\right)}^{2}+{\left(\frac{\dot{x_{D}}-\dot{y_{D}}}{2}\right)}^{2}\right]$$

where $m_{B_{D1}}$ is the mass of the part $B_{D1}$, $\lambda_{1}$ is the rotation compensation coefficient.

$m_{B_{D1}}$ can be expressed as:

$$m_{B_{D1}}=L_{B_{D1}}w_{B_{D1}}b\rho +\left[2\left(2R+t\right)R-\pi R^{2}\right]b\rho$$

As shown in Figure 15, the kinetic energy of the unit $C_{D}$ can be expressed as:

(16) $$T_{C_{D1}}=\frac{1}{2}m_{C_{D1}}\left[{\left(\frac{\dot{x_{D}}+\dot{y_{D}}}{2}\right)}^{2}+{\left(\frac{\dot{x_{D}}-\dot{y_{D}}}{2}\right)}^{2}\right]$$

where $m_{C_{D1}}$ is the mass of the unit $C_{D1}$.

**Figure 18:**
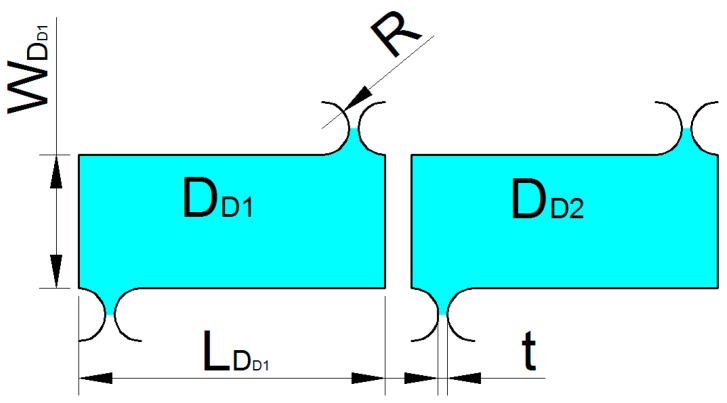
Unit $D_{D}$ structure.

As shown in Figure 18, since the motion of the unit $D_{D}$ is also extremely complicated, this paper only considers the translational motion. Therefore, the kinetic energy of the unit $D_{D1}$ can be expressed as:

(17) $$T_{D_{D1}}=\frac{1}{2}m_{D_{D1}}\left[\lambda_{2}{\left(\frac{\dot{x_{D}}+\dot{y_{D}}}{2}\right)}^{2}+{\left(\frac{\dot{x_{D}}-\dot{y_{D}}}{2}\right)}^{2}\right]$$

where $m_{D_{D1}}$ is the mass of the part $D_{D1}$, $\lambda_{2}$ is the rotation compensation coefficient.

$m_{D_{D1}}$ can be expressed as:

(18) $$m_{D_{D1}}=L_{D_{D1}}w_{D_{D1}}b\rho +\left[2\left(2R+t\right)R-\pi R^{2}\right]b\rho$$

**Figure 19:**
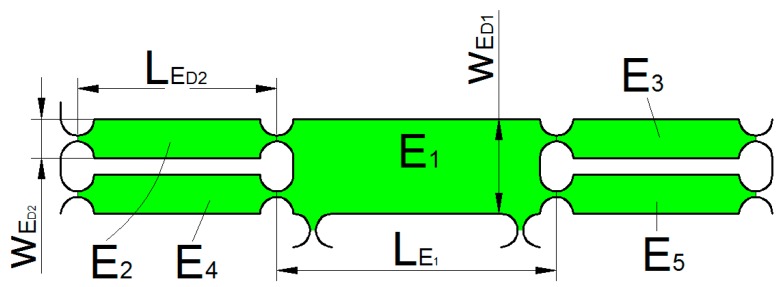
Unit $E_{D}$ structure.

As shown in Figure 19, $E_{D}$ can be divided into five parts of $E_{D1}$~$E_{D5}$.

The kinetic energy of the unit $E_{D1}$ can be expressed as:

(19) $$T_{E_{D1}}=\frac{1}{2}m_{E_{D1}}{\dot{y_{D}}}^{2}$$

where $\dot{y_{D}}$ is the speed of the unit $E_{D1}$, $m_{E_{D1}}$ is the mass of the unit $E_{D1}$.

$m_{E_{D1}}$ can be expressed as:

(20) $$m_{E_{D1}}=(L_{E_{D1}}-2R)w_{E_{D1}}b\rho +3\left[2w_{E_{D2}}R-\pi R^{2}\right]b\rho$$

The kinetic energy of the part $E_{D2}$ can be expressed as:

(21) $$T_{E_{D2}}=\frac{1}{2}I_{E_{D2}}{\left(\frac{\dot{y}}{L_{E_{D2}}}\right)}^{2}$$

where $I_{E_{D2}}$ is the rotational inertia of the unit $E_{D2}$.

$I_{E_{D2}}$ can be expressed as:

(22) $$I_{E_{D2}}=m_{E_{D2}}\left[\frac{1}{3}{\left(L_{E_{D2}}-2R\right)}^{2}+(L_{E_{D2}}-2R\right)]R+R^{2}]$$

where $m_{E_{D2}}$ is the mass of the part $E_{D2}$.

$m_{E_{D2}}$ can be expressed as:

(23) $$m_{E_{D2}}=(L_{E_{D2}}w_{E_{D2}}-\pi R^{2})b\rho$$

**Figure 20:**
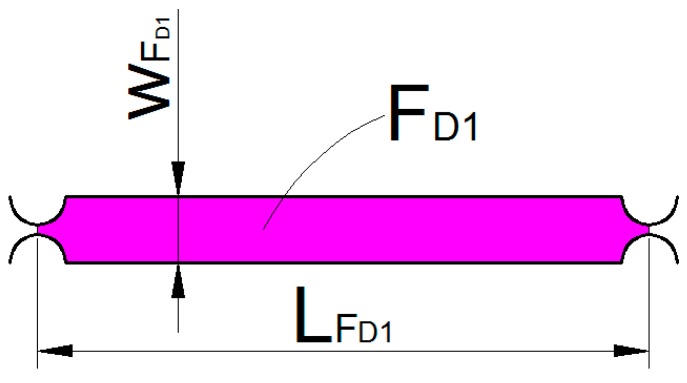
Unit $F_{D}$ structure.

As shown in Figure 20, $F_{D}$ can be divided into eight parts of $F_{D1}$~$F_{D8}$. The kinetic energy of the part $F_{D1}$ can be expressed as:

(24) $$T_{F_{D1}}=\frac{1}{2}m_{F_{D1}}{\left(\frac{\dot{x_{D}}-\dot{y_{D}}}{2}\right)}^{2}+\frac{1}{2}I_{F_{D1}}{\left(\frac{\dot{x_{D}}+\dot{y_{D}}}{2L_{F_{D1}}}\right)}^{2}$$

where $m_{F_{D1}}$ is the mass of the part $F_{D1}$, $I_{F_{D1}}$ is the rotational inertia of the unit $F_{D1}$.

$m_{F_{D1}}$ can be expressed as:

$$m_{F_{D1}}=(L_{F_{D1}}w_{F_{D1}}-\pi R^{2})b\rho$$

**Figure 21:**
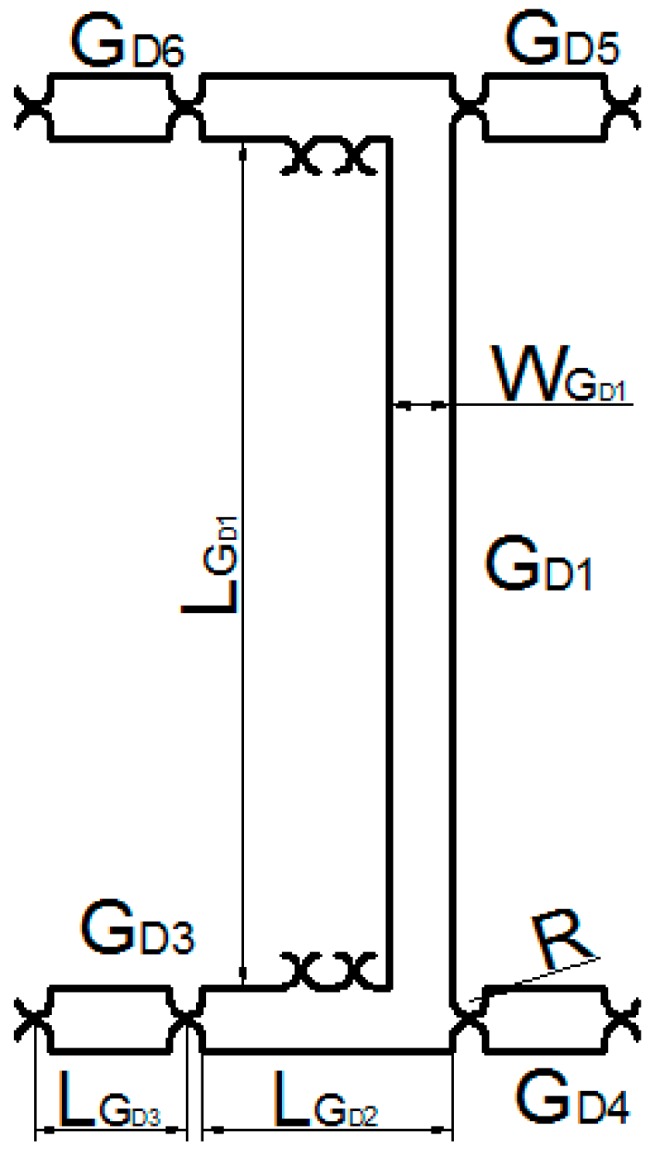
Unit $G_{D}$ structure.

As shown in Figure 21, $G_{D}$ can be divided into ten parts of $G_{D1}$~$G_{D10}$. The kinetic energy of the part $G_{D1}$ can be expressed as:

(25) $$T_{G_{D1}}=\frac{1}{2}m_{G_{D1}}{\left(\frac{\dot{x_{D}}-\dot{y_{D}}}{2}\right)}^{2}$$

where $m_{G_{D1}}$ is the mass of the part $G_{D1}$.

$m_{G_{D1}}$ can be expressed as:

(26) $$m_{G_{D1}}=L_{G_{D1}}w_{G_{D1}}b\rho +L_{G_{D2}}w_{G_{D1}}b\rho +3\left(2w_{G_{D1}}R-\pi R^{2}\right)b\rho$$

The kinetic energy of the part $G_{D2}$ can be expressed as:

(27) $$T_{G_{D2}}=\frac{1}{2}I_{G_{D2}}{\left(\frac{\dot{x_{D}}-\dot{y_{D}}}{2L_{G_{D3}}}\right)}^{2}$$

where $I_{G_{D2}}$ is the rotational inertia of the unit $G_{D2}$.

$I_{G_{D2}}$ can be expressed as:

(28) $$I_{G_{D2}}=m_{G_{D2}}\left[\frac{1}{3}{\left(L_{G_{D3}}-2R\right)}^{2}+(L_{G_{D3}}-2R\right)]R+R^{2}]$$

$m_{G_{D2}}$ can be expressed as:

(29) $$m_{G_{D2}}=(L_{G_{D3}}w_{G_{D2}}-\pi R^{2})b\rho$$

According to Equations (9)–(29), the total kinetic energy of the device can be expressed as

(30) $$T=T_{A_{D1}}+4T_{A_{D2}}+2T_{B_{D1}}+T_{C_{D1}}+2T_{D_{D1}}+T_{E_{D1}}+4T_{E_{D2}}+8T_{F_{D1}}+2T_{G_{D1}}+8T_{G_{D2}}$$

Equation (30) can be described as:

(31) $$T=0.5M_{1}{\dot{x_{D}}}^{2}+0.5M_{2}{\dot{y_{D}}}^{2}$$

According to Equation (31), the quality matrix is

(32) $$M=\left[\begin{array}{c} M_{1}\;\;\;\;0\\ \;\;0\;\;\;\;\;\;M_{2} \end{array}\right]$$

where *M*_1_ is the equivalent quality matrix of *x*, *M*_2_ is the equivalent quality matrix of *y*.

The stiffness matrices can be obtained according to the results of static investigation, and can be expressed as:

(33) $$K=\left[\begin{array}{c} K_{3}\;\;\;\;0\\ \;\;0\;\;\;\;\;\;K_{4} \end{array}\right]$$

where *K*_3_ is the stiffness of the No. 1 piezoelectric input terminal, *K*_4_ is the stiffness of the No. 2 piezoelectric input terminal.

Then the first natural frequency and the second natural frequency can be calculated.

(34) $$f=\frac{1}{2\pi }\sqrt{\frac{K}{M}}$$

Using the energy method, the first natural frequency is 439.59 Hz and the second natural frequency is 690.29 Hz.

#### 3.2.2. Finite Element Analysis (FEA)

**Figure 22:**
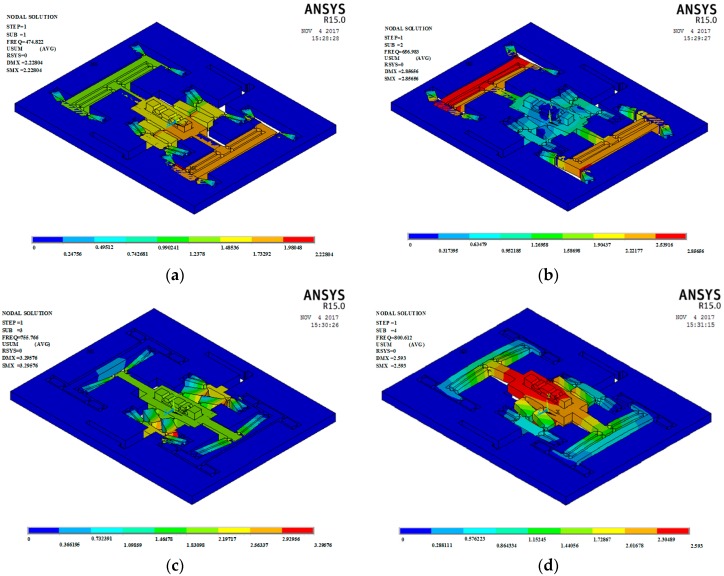
Modal analysis of 3-DOF device. (**a**) Mode 1; (**b**) Mode 2; (**c**) Mode 3; (**d**) Mode 4.

In order to verify the natural frequency calculated by the energy method, the FEA method was used for modal analysis. The full constraint was applied to the four boundaries in the model. Solid model 10 node 187 elements were used in finite element model. The material is aluminum alloy with elasticity, linearity and isotropy. The elastic modulus is 70 MPa. Poisson’s ratio is 0.3. The material density is 2.78 g/cm^3^. Figure 22 gives the vibrational mode shapes from mode 1 to mode 4. The natural frequencies of the first to fourth are 474.82 Hz, 656.98 Hz, 755.78 Hz and 800.61 Hz, respectively. For the first and second natural frequencies, the relative deviations of FEA and energy method are 7.4196% and 4.8255% respectively. This shows that the results of the FEA method are more or less consistent with the results of the energy method.

## 4. Device Performance Test

### 4.1. Natural Frequency

**Figure 23:**
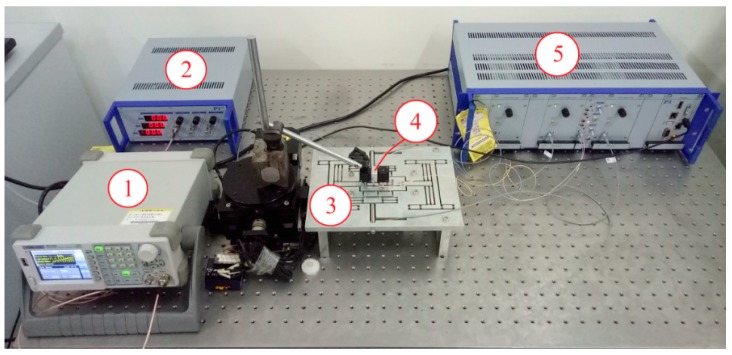
Natural frequency detection system. (**1**) signal generator; (**2**) power amplifier; (**3**) three-dimensional (3D) motion device; (**4**) capacitance sensor; (**5**) capacitance detection channel.

**Figure 24:**
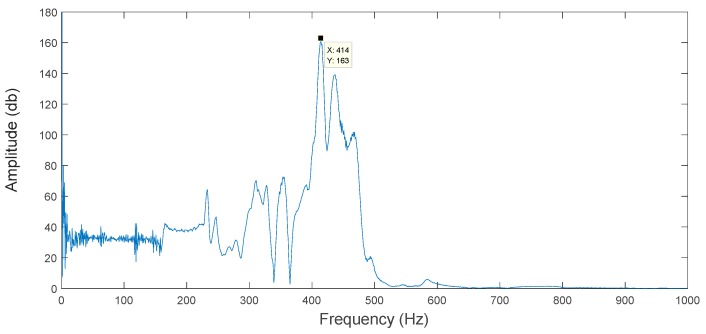
Amplitude-frequency curve of a 3-DOF motion device.

In this paper, a non-resonant 3-DOF motion device is designed. In order to maintain the stability of the device and avoid damage, its operating frequency should effectively avoid the device’s natural frequency. In this paper, the natural frequency of the device was detected by the sweeping frequency method. The test system is shown in Figure 23. FEA shows that the direction of vibration of the first natural frequency is the *y* direction. A sine sweep signal (voltage range: 0.5–2 V, frequency range: 0–1000 Hz) is generated by a signal generator (Brand: Siglent Model: Sdg805, Shanghai, China.) and applied to a No. 1 piezoelectric actuator (Brand: Physik Instrumente (PI) GmbH Model: P-820.20, Karlsruhe, Germany.) of a 3D motion device via a power amplifier (Brand: Physik Instrumente (PI) GmbH Model: E-663.00, Karlsruhe, Germany.). The capacitive sensor (Brand: Physik Instrumente (PI) GmbH Model: D-100.00, Karlsruhe, Germany.) is used to measure the output response in each direction, and the capacitance detection module (Brand: Physik Instrumente (PI) GmbH Model: PI E509, Karlsruhe, Germany.) and the communication module (Brand: Physik Instrumente (PI) GmbH Model: PI E516, Karlsruhe, Germany.) are used to achieve the output signal acquisition in each direction. The *y*-direction output response curve obtained by Fourier transform is shown in Figure 24. For the resonance frequency curve, the highest point of the curve is the first natural frequency of the motion device. Observing the response curve, the first natural frequency of the experiment is 414 Hz. The natural frequencies of the energy method and the FEA are 439.59 Hz and 474.82 Hz, respectively. The experimental natural frequency is slightly lower than the theoretical analysis. The main reason is that the assembly of the device is non-rigid and the capacitive sensor is mounted at the output, which improves the motion quality of the device.

### 4.2. Dynamic Performance

**Figure 25:**
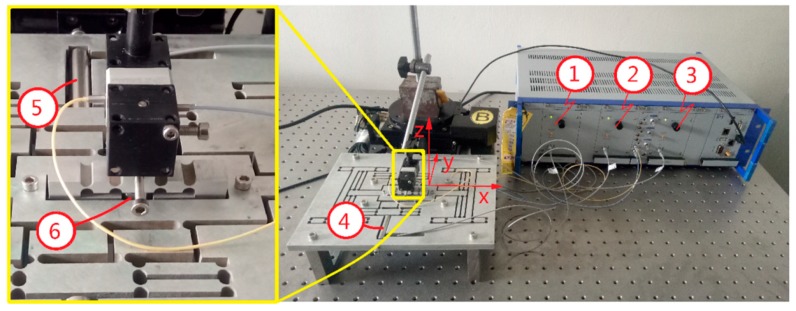
Experiment of device response performance test.

**Figure 26:**
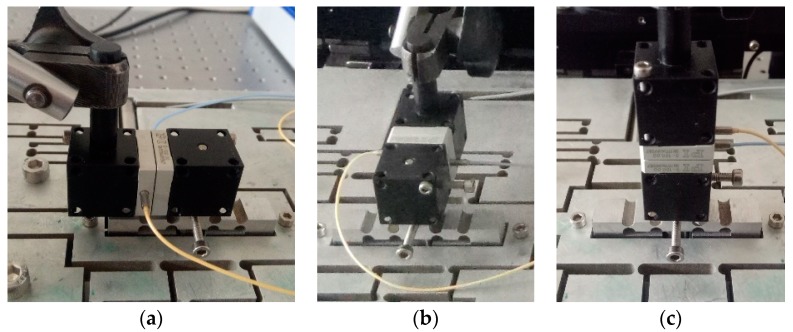
Capacitive sensor installation in all directions. (**a**) *x*-direction test; (**b**) *y*-direction test; (**c**) *z*-direction test.

In general, if the mechanism can adapt to the stair step signal, the mechanism can meet the dynamic performance requirements of other signals. In order to simplify the experimental test, a square wave signal is used instead of a stair step signal. Sinusoidal signal is the most basic input signal of 3-DOF motion device and is widely used in engineering. However, the mechanism has a return trip effect. In order to better detect the output response of the device, triangle wave and sine wave are selected as test signals in this paper. The flexible mechanism performance test is shown in Figure 25 and Figure 26. In Figure 25, ➀, ➁, ➂ are power amplifiers connected to the No. 1, No. 2 and No. 3 piezoelectric actuators, respectively; ➃, ➄, ➅ are No. 1, No. 2 and No. 3 piezoelectric actuators, respectively. The capacitive sensor installed in the *x*, *y* and *z* directions is shown in Figure 26. In the experiment, the capacitance sensor moving plate is installed at the output terminal of the three-dimensional motion device, and the fixed plate is installed on the fixed frame. When the signal generator output signal is unchanged, the *x*, *y*, and *z* directions are tested to obtain a distance curve in each direction. (The distance data measured by the experimental system begin with the same phase of the input signal.) The performance curves of the output terminal of the device are obtained by comprehensive analysis.

(1)   *x*-direction

**Figure 27:**
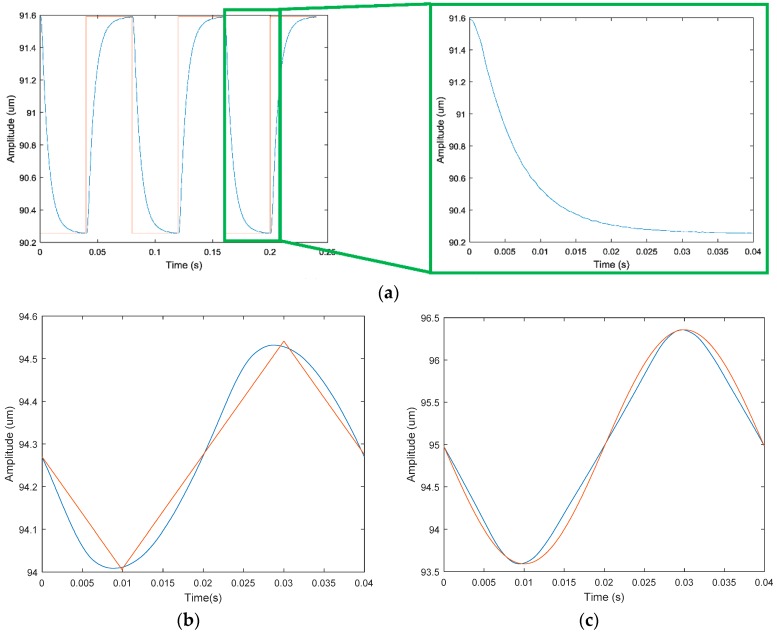
Response curve in *x* direction. (**a**) Square wave signal; (**b**) triangle wave signal; (**c**) sine wave signal.

The square wave signals with the same frequency and phase were applied to the No. 1 and No. 2 piezoelectric actuators. Theoretically, square wave signals of the same period should be produced in the *x* direction. The response curve of the output terminal in the *x* direction is shown in Figure 27a. Observing the test curve for a half cycle, the response displacement is exponentially decayed. After 0.025 s, the output signal tends to be flat because the return response of the piezoelectric system and mechanism is lagging. The triangular wave and sine wave signals of the same phase and the same frequency were applied to the No. 1 piezoelectric actuator and No. 2 piezoelectric actuator, respectively. The output curves of the system are shown in Figure 27b,c. The sinusoidal response of the system is better, while the square wave and triangle wave response is poor.

(2)   *y*-direction

**Figure 28:**
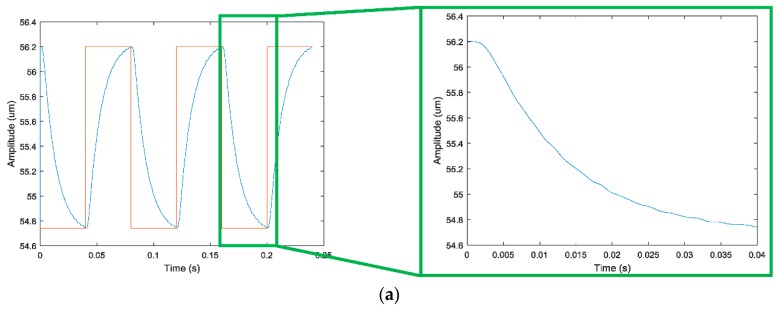

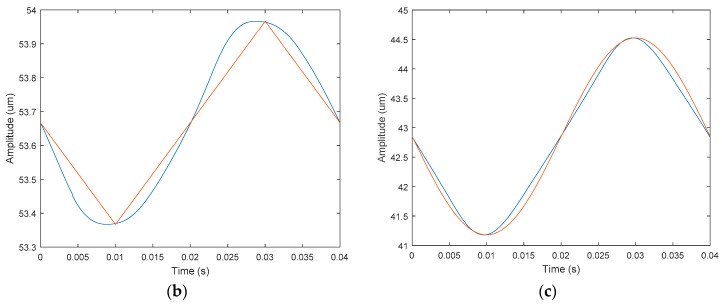
Response curve in *y* direction. (**a**) Square wave signal; (**b**) triangle wave signal; (**c**) sine wave signal.

The square wave, triangle wave, and sinusoidal signals with the same frequency and a phase difference of 180° were applied to the No. 1 piezoelectric actuator and No. 2 piezoelectric actuator. The response curve of the output terminal in the *y* direction is shown in Figure 28. The output response curve in the *y* direction was observed and it was found that: (1) When the rectangular square wave signal is used as the input signal, at 0.025 s, the output terminal responds to 90% of the theoretical output response; (2) when the triangular wave signal is used as the input signal, the output signal amplitude fluctuates around the theoretical output signal; (3) when the sine wave signal is used as the input signal, the output signal is basically the same as the theoretical output signal.

(3)   *z*-direction

**Figure 29:**
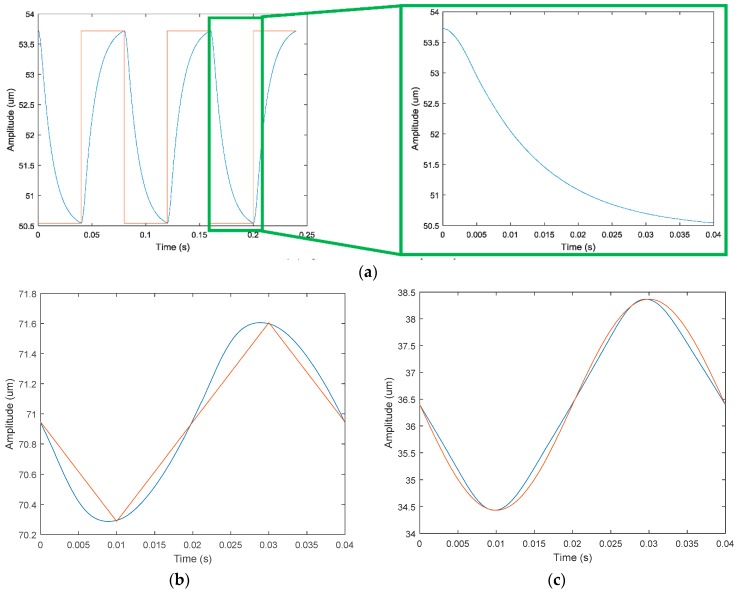
Response curve in *z* direction. (**a**) Square wave signal; (**b**) triangle wave signal; (**c**) sine wave signal.

The square rectangular, triangular, and sinusoidal signals are applied to the No. 3 piezoelectric actuator. The response curve of the output terminal in the *z* direction is shown in Figure 29. The output response curve in the *z* direction was observed and found that: (1) when the rectangular square wave signal is an input signal, at 0.027 s, the output terminal responds to 90% of the theoretical output response; (2) when the triangular wave signal is an input signal, the output signal amplitude fluctuates around the theoretical output signal; (3) when the sine wave signal is an input signal, the output signal is more or less the same as the theoretical output signal.

### 4.3. Linear Motion Performance in Each Direction

Although the 3-DOF device can theoretically actualize independent movements in the *x*, *y* and *z* directions, there may be a discrepancy between the actual displacement and the theoretical displacement. In order to verify the linear motion ability, three input signals that theoretically produce only *x*, *y* and *z* directions movements are applied to the device.

**Figure 30:**
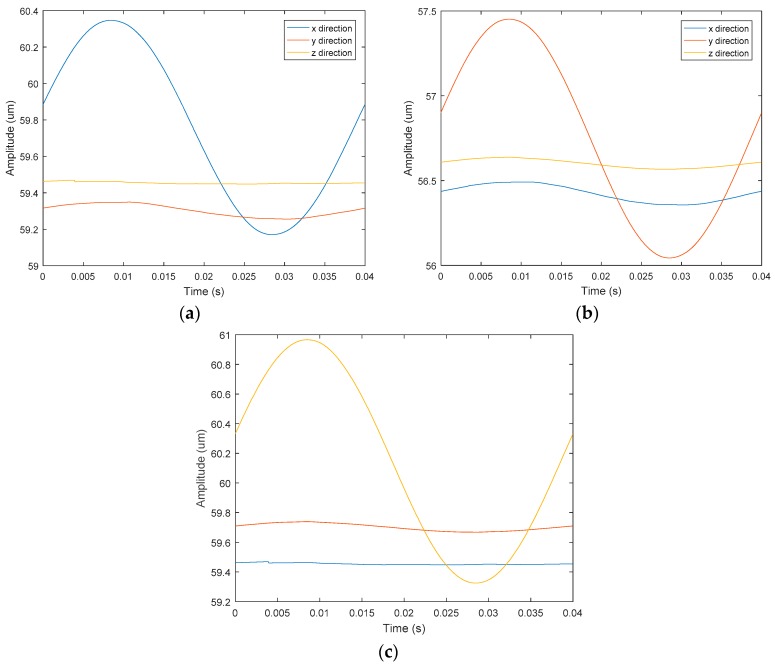
Time-amplitude diagram of linear motion performance test in each direction. (**a**) Driven in *x* direction; (**b**) driven in y direction; (**c**) driven in *z* direction.

The sinusoidal signals with the same frequency and phase were applied to the No. 1 and No. 2 piezoelectric actuators. Theoretically, the device only produces *x*-direction displacement. The output displacement of the three directions, *x*, *y* and *z*, were detected, respectively. The relationship between displacement and time in three directions is shown in Figure 30a. The following conclusions were drawn: The amplitude of the *x* direction is much larger than the *y* and *z* directions, the *y* direction response is sinusoidal, and the amplitude is significantly larger than the *z* direction response amplitude.

**Figure 31:**
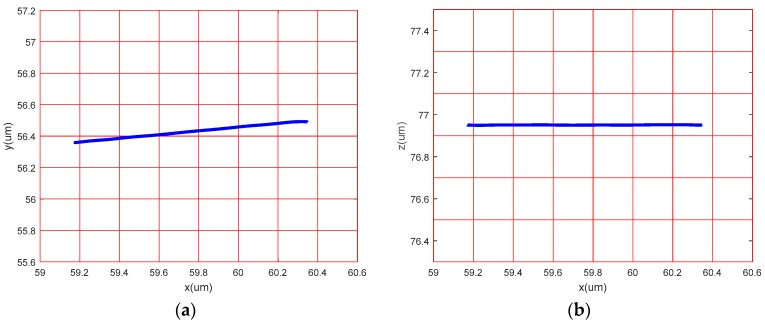
Linear motion performance test in *x* direction. (**a**) Projection on the *xoy* plane; (**b**) projection on the *xoz* plane.

**Figure 32:**
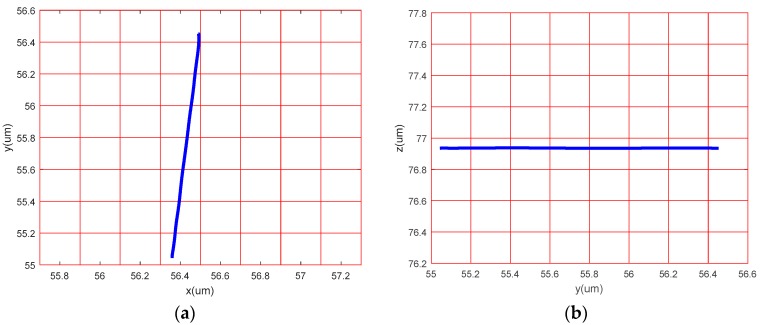
Linear motion performance test in *y* direction. (**a**) Projection on the *xoy* plane; (**b**) projection on the *yoz* plane.

**Figure 33:**
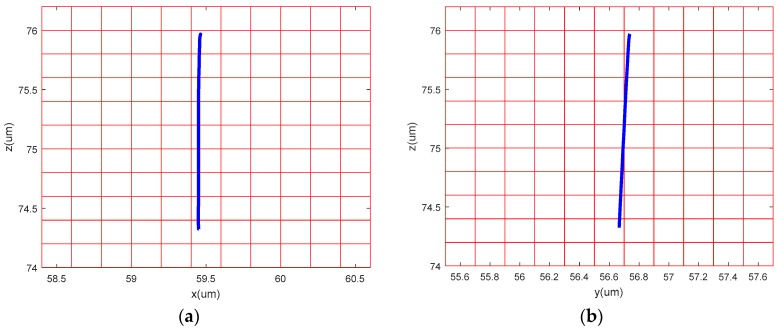
Linear motion performance test in *z* direction. (**a**) Projection on the *xoz* plane; (**b**) projection on the *yoz* plane.

The sinusoidal signals with the same frequency and a phase difference of 180° were applied to the No. 1 piezoelectric actuator and No. 2 piezoelectric actuator, respectively. Theoretically, the device only produces *y* direction displacement. The output displacement of the three directions, *x*, *y* and z, was detected respectively, as shown in Figure 30b. The sinusoidal signals were applied to the No. 3 piezoelectric actuator and the output displacements of the three directions, *x y* and *z,* were detected, as shown in Figure 30c. The experimental curve shows that the device has large deviations from the actual operation and theoretical operation in the *x* direction and the *y* direction. Figure 31, Figure 32 and Figure 33 show the projected trajectories of linear motion along the *x*, *y* and *z* axes.

Table 2 provides some data from the linear motion performance test. The maximum linear motion deviations in the *x*, *y* and *z* directions were approximately 6.67%, 5.71%, and 3.03%, respectively. The reasons for this error are mainly because of the following aspects: (1) since the mechanical midpoint of the power component of the device is not exactly the same as the mechanical midpoint of the *x*, *y* output decoupling guiding element, the output block is slightly deflected; (2) the installation error has a great influence on the experimental results; (3) the inconsistent piezoelectric preload may cause the linear motion performance to deteriorate in *x* and *y* directions.

### 4.4. 3D Vibration Trajectory

**Figure 34:**
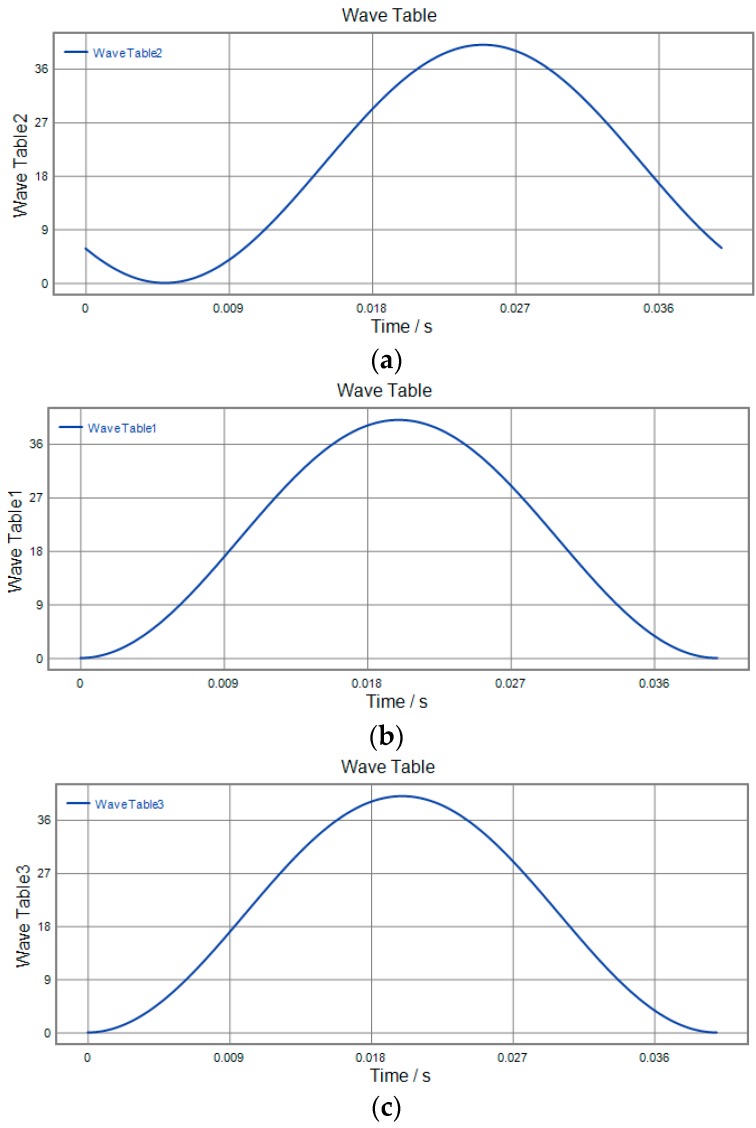

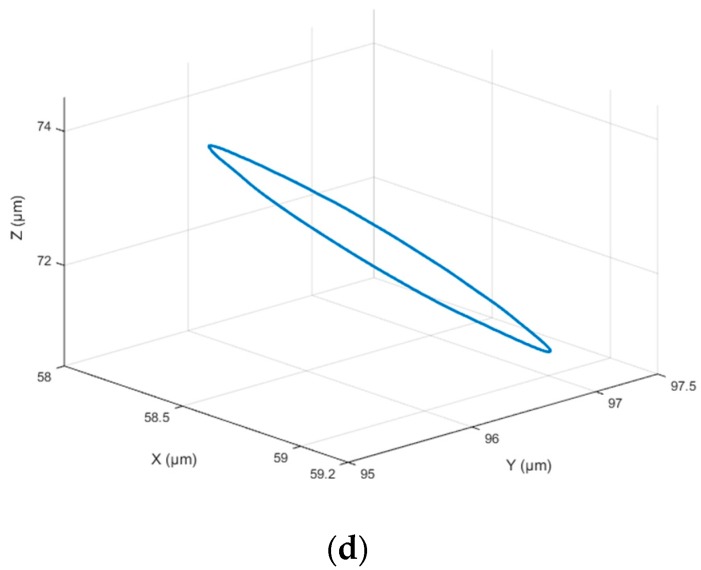
Experimental scenario 1. (**a**) *x*-direction input signal; (**b**) *y*-direction input signal; (**c**) *z*-direction input signal; (**d**) output trajectory.

**Figure 35:**
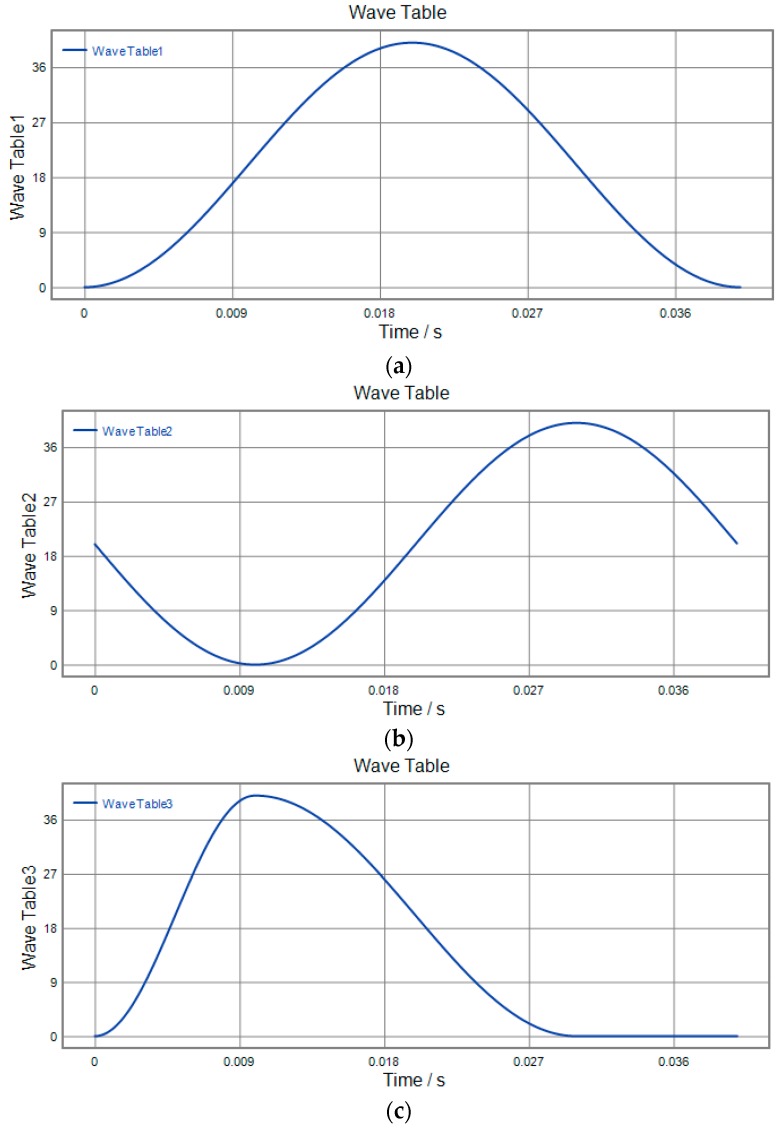

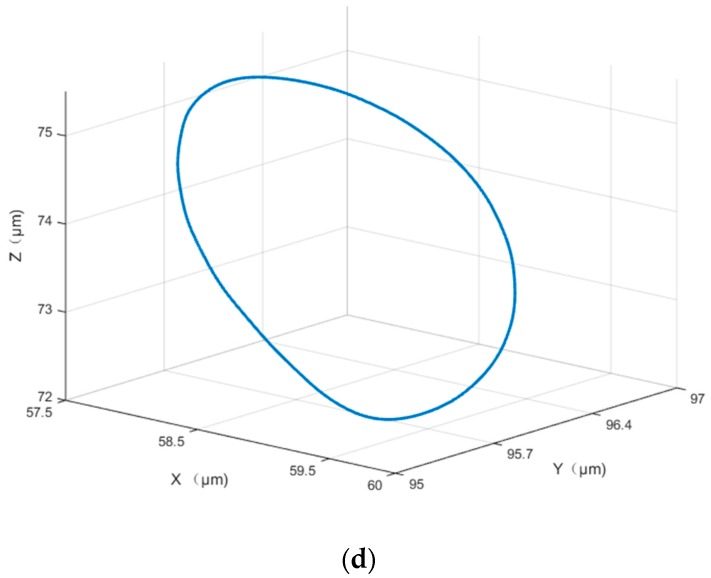
Experimental scenario 2. (**a**) *x*-direction input signal; (**b**) *y*-direction input signal; (**c**) *z*-direction input signal; (**d**) output trajectory.

**Figure 36:**
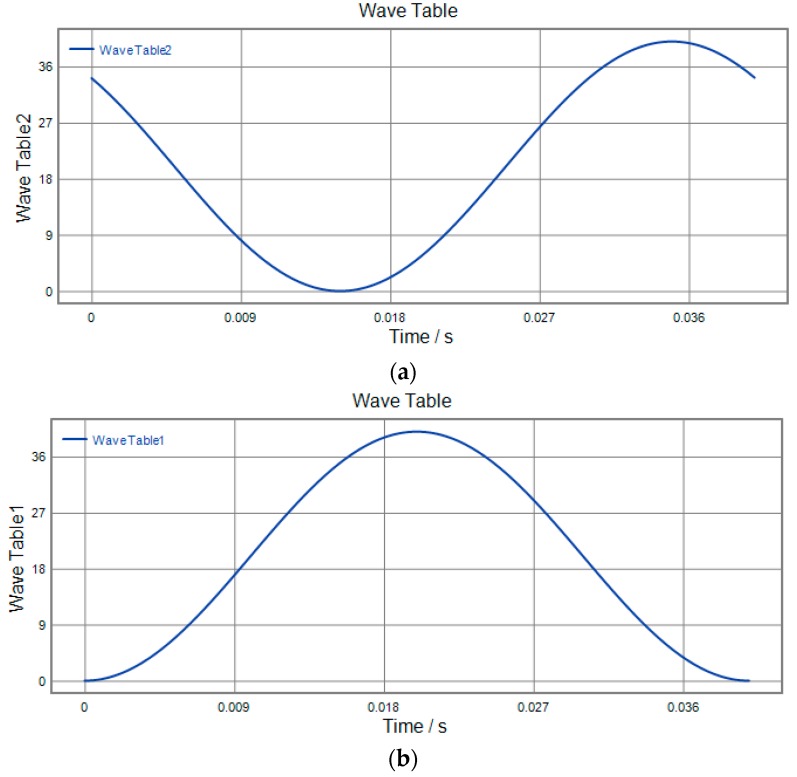

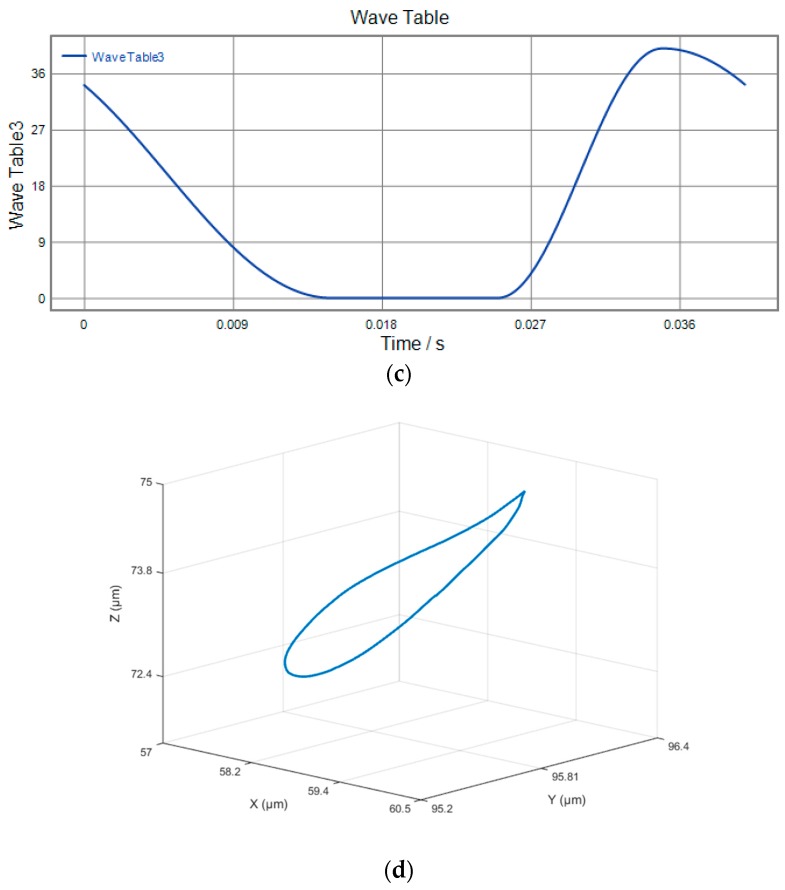
Experimental scenario 3. (**a**) *x*-direction input signal; (**b**) *y*-direction input signal; (**c**) *z*-direction input signal; (**d**) output trajectory.

**Figure 37:**
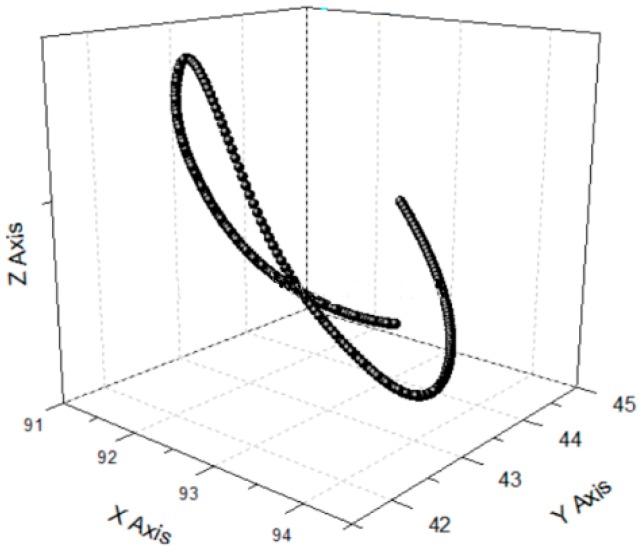
Output trace No. 1.

**Figure 38:**
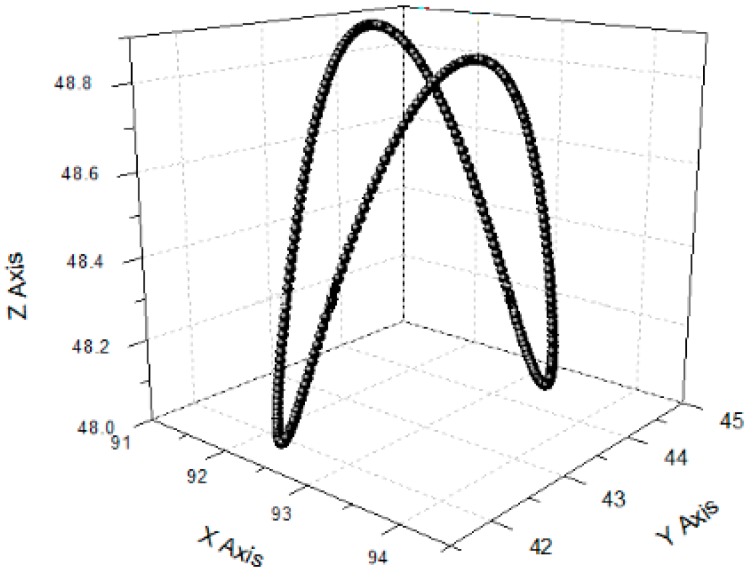
Output trace No. 2.

3D vibration device can realize parallel movement in the *x*, *y* and *z* directions. It is necessary that the three directions of *x y* and *z* cooperate to realize the 3D movement of the output terminal against actual application. In order to study the actual movement of the output terminal, different signals are applied to the No. 1, No. 2 and No. 3 piezoelectric actuators, respectively. The motion trajectory of the device in all directions is detected using a capacitive sensor. The actual running trajectory is synthesized in a 3D space by detecting the displacement of *x*, *y*, *z* to the output during one cycle. The three experimental schemes are shown in Figure 34, Figure 35 and Figure 36 respectively, and the running trajectories are shown in Figure 34d, Figure 35d and Figure 36d. In addition, the actual operational trajectory of the device is shown in Figure 37 and Figure 38, when more complex signals are used as inputs.

## 5. Application Test

**Figure 39:**
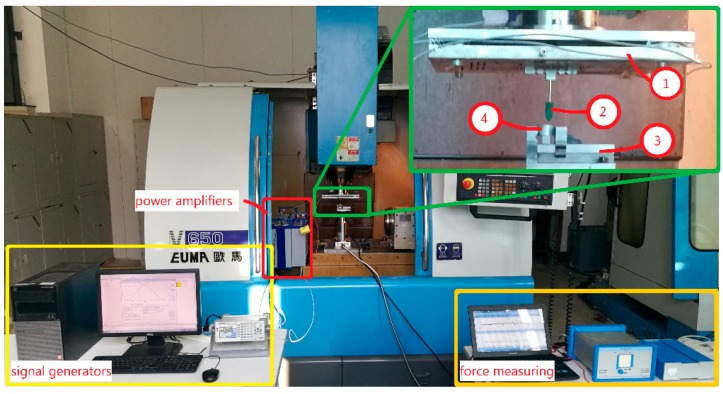
3D vibration scratching experiment system (**1**) flexible mechanism device; (**2**) experimental tool; (**3**) workpiece holder; (**4**) workpiece.

**Figure 40:**
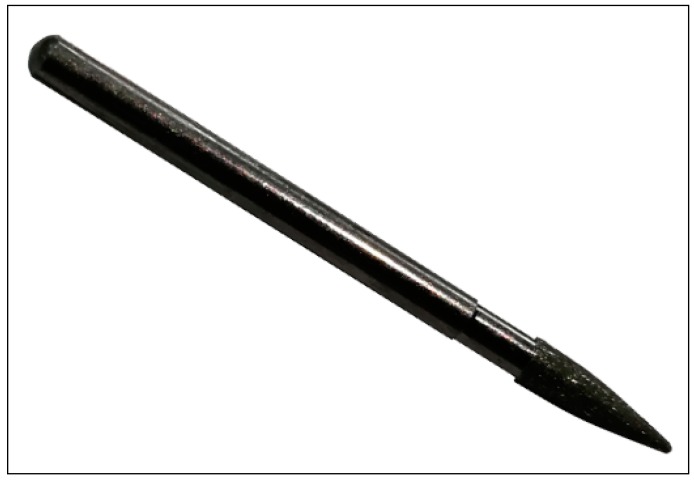
Experimental tool.

The scratching experiment system is shown in Figure 39 and the experimental tool is shown in Figure 40. Since the output response of the device under no-load conditions is significantly different from the output response of the load, this still needs to be tested. There are two reasons that account for this. On the one hand, the piezoelectric actuators will have a smaller output displacement when the output terminal is subjected to external loads compared to no-load. On the other hand, it is possible to cause internal deformation without generating an output displacement in a complex mechanical environment, if the design of the device is unreasonable. Therefore, two experiments are described in the following section to observe the actual effect of the device under complex working conditions. The flexible mechanism device was installed on the spindle. The *y*-feed of the machine tool and the 3D vibrations jointly complete the scratching experiment. Table 3 gives the signal parameters applied to the motion device. The signal in *x* and *y* directions is generated by the piezoelectric driving control system. The signal in *z* direction is generated by the signal generator. When the three directions’ signals are amplified by the power amplifier and applied to the No. 1, No. 2 and No. 3 piezoelectric actuators, respectively, flexible mechanism devices can produce 3D movement in space. Two groups of scratch experiments were carried out. A workpiece (material: aluminum alloy, AISI6061) is fixed to the machine tool with a clamp and the tool is fixed at the 3D motion device output terminal. The *y*-feed rate of the machine table is 2 mm/min. The force measuring system (Brand: kistler Model: 9129AA, Winterthur, Switzerland.) is installed on the bottom of the workpiece holder in order to detect the three directions’ polishing force of the workpiece. The surface topography of the workpiece was observed using a microscope (Brand: Olympus Model: BX-15, Tokyo, Japan.) after the scratch experiment.

**Figure 41:**
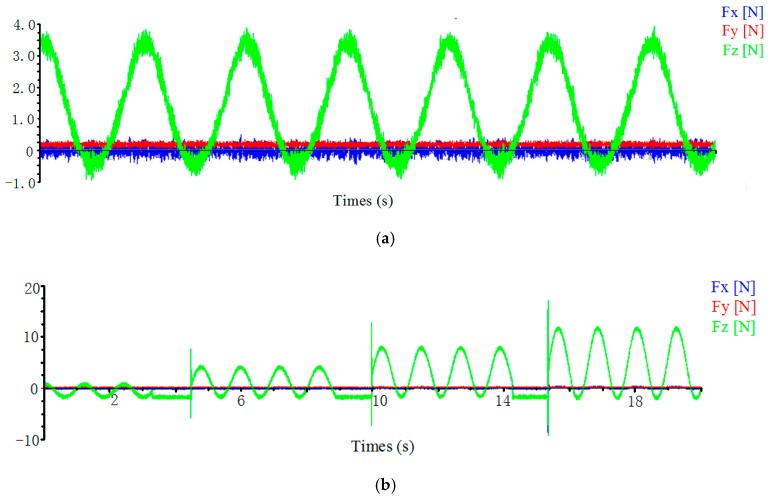
Force curve. (**a**) Effect of *z*-direction vibration on initial force; (**b**) Force curve during the experiment.

**Figure 42:**
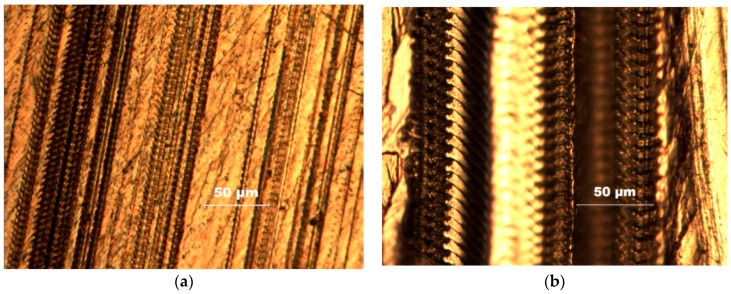
Workpiece surface topography. (**a**) Experiment No. 1; (**b**) Experiment No. 2.

The green rubber tool was used in Experiment No.1. When the sine voltage signal is applied to three directions of flexible mechanism device, the force curve of the workpiece is shown in Figure 41a and the workpiece surface obtained by the experiment is shown in Figure 42a. The diamond tool was used in Experiment No. 2. When a sinusoidal voltage signal of 2, 4, 6, and 8 V is applied to *z*-direction of flexible mechanism device respectively, the force curve of the workpiece is shown in Figure 41b. When an 8 V voltage signal was used as an input, the workpiece surface obtained by the experiment is as shown in Figure 42b. Based on Figure 41, it is concluded that *z*-direction vibration has the greatest influence on the force of the workpiece. The reason is that the polishing tool is perpendicular to the surface of the workpiece in the z direction, so the *z*-direction force changes very significantly; the *x*-direction force changes is second. While the *y*-direction is the actual tool feed direction, which is affected by the feed, its force change curve amplitude is small; the *z*-direction force increases as the amplitude of the voltage signal increases; Figure 42 shows the surface micro-morphology of the scratched workpiece and it was found that the workpiece surface has a clear, periodic microstructure. When the *z*-direction operating cycle of the 3D motion device is increased by four times, the number of microstructures is reduced to 1/4. This is an effective basis for verifying the practical performance of the device.

## 6. Conclusions

In this paper, a 3-DOF motion device based on a piezoelectric actuated flexible mechanism was designed, which has the characteristics of simple control, high output stiffness and self-guided output. Theoretical analysis, FEA, performance testing and experiments were carried out. The main conclusions are as follows:

1. The device is mainly series connected by a 2D motion platform including the *x* and *y* directions, and the independent movement structure with *z* direction. The 3-DOF device adopts a circular flexure hinge, in which the 2D motion platform consists of three parts: driving element, force-dividing element (composed of four force-dividing blocks), x and y output decoupling guide element. The driving element is designed using the principle of double parallelograms. The function of the force-dividing element is to decompose the force into *x* and *y* directions; The x and y output decoupling guide element can improve the output stiffness, and running accuracy, and detect the actual displacement in the *x* and *y* directions.
2. The statics and dynamics of the 3-DOF motion device were analyzed. Using the MCM method, the output stiffness in the *x*, *y* and *z* directions are 1.137 N/μm, 0.760 N/μm and 0.717 N/μm. Using the FEA method, the output stiffness in the *x*, *y* and *z* directions are 1.178 N/μm, 0.751 N/μm, and 0.659 N/μm. Using energy method, the first natural frequency is 439.59 Hz and the second natural frequency is 690.29 Hz. Using FEA method, the first natural frequency is 474.82 Hz and the second natural frequency is 656.98 Hz. The first natural frequency of the experiment is 414 Hz.
3. The device was tested for dynamic performance, linear motion, and vibration trace during no-load. Square wave, triangle wave and sine wave signals were applied in three directions to test dynamic response performance. When the square wave is input, the *x*-direction return response basically reaches the theoretical operating position at 0.025 *s*; the *y* direction and *z* direction reach 90% of the theoretical operating position at 0.025 s and 0.027 s, respectively. When the triangular wave signal is an input signal, the output signal amplitude fluctuates around the theoretical output signal; when the sine wave signal is an input signal, the output signal is basically the same as the theoretical output signal. When a signal that can only generate unidirectional motion is applied, the maximum linear motion errors in the *x*, *y* and *z* directions are approximately 6.67%, 5.71%, and 3.03%. Different signals were applied to the No. 1, No. 2 and No. 3 piezoelectric actuators to carry out five sets of vibration track test experiments. These results show that the device has excellent dynamic performance and can achieve 3D spatial trajectory.
4. A three-dimensional vibration scratch experiment was carried out using a 3D motion device. When the vibration signal is only applied to the 3-DOF motion device, since the polishing tool is perpendicular to the surface of the workpiece in the *z* direction, the *z*-direction exhibits a significant periodic force change and coincides with the *z*-direction output signal cycle. The period and shape of the force curve are similar when the table is feeding or stationary. The workpiece surface scratched presents a pronounced periodic structure. These results proved the 3-DOF motion device has better reliability.

## Figures and Tables

**Table 1:** The comparison of output compliance.

Methods	F_X_ (N)	δX (μm)	Error (%)	F_Y_ (N)	δY (μm)	Error (%)	F_Z_ (N)	δZ (μm)	Error (%)
FEA	100	84.87	3.612	100	133.17	1.156	100	151.86	8.126
MCM	100	87.94		100	131.63		100	139.52	

**Table 2:** Experimental data analysis of linear motion performance.

Project	Maximum Displacement		
	*x* Direction (μm)	*y* Direction (μm)	*z* Direction (μm)
*x* direction input signal	1.2	0.08	0.03
*y* direction input signal	0.08	1.4	0.05
*z* direction input signal	0.01	0.01	1.65

**Table 3:** Experimental signal parameters.

Project	Tool	Sinusoidal Signal Direction	Low Voltage (V)	High Voltage (V)	Cycle (s)	Frequency Difference between *x* and *y* (s)
Experiment No. 1	Green rubber tool	*x*	0	8	0.006	0.001
		*y*	0	8	0.006	
		*z*	0	8	0.3	
Experiment No. 2	Diamond tool	*x*	0	8	0.006	0.001
		*y*	0	8	0.006	
		*z*	0	8	1.2	

## Supplementary Materials

The following are available online at http://www.mdpi.com/2072-666X/9/11/578/s1, Figure S1: Structure and driving principle of force-dividing block, Figure S2: Force-dividing block input-output response, Figure S3: The displacement nephogram of FEA, Figure S4: 3D motion device structure. (**a**) 3D motion device structural solid model; (**b**) Structural combination model, Figure S5: Schematic diagram of output center error position, Figure S6: Circular flexure hinge, Figure S7: Beam flexure hinge, Table S1: The structure parameters and FEA results, Table S2: Correction coefficient *k*.
