# Supplementary material for: Research on a 3-DOF Motion Device Based on the Flexible Mechanism Driven by the Piezoelectric Actuators
Source: Micromachines (Basel). 2018 Nov 6;9(11):578. doi: 10.3390/mi9110578 (PMC6265935; doi:10.3390/mi9110578)
Supplement: Supplementary file 1 [file micromachines-09-00578-s001.pdf]

Supplementary Materials: Research on a 3-DOF Motion Device Based on the Flexible Mechanism Driven by the Piezoelectric Actuators

Bingrui Lv, Guilian Wang *, Bin Li, Haibo Zhou and Yahui Hu

S1. Analysis of x-Direction Drive Displacement

The force-dividing block is shown in Figure S1. When the compression deformation of the force-dividing block is not considered, the force-dividing block length can be expressed as:

$$l = \sqrt{x^2 + y^2} \tag{S1}$$

When the 2D motion platform output block moves along the *x*-axis, the No. 1 hinge moves in the *y* direction and the No. 2 hinge moves in the *x* direction. Assuming that the No. 1 hinge is moved y_3 in the *y* direction, at this time, the No. 2 hinge is moved x_3 along the *x* direction. The parameter relationship between x_3 and y_3 is obtained as follows:

$$y_3 = y - \sqrt{l^2 - (x + x_3)^2} \tag{S2}$$

According to Equations (S1) and (S2), when *x*=18.3 *mm* and *y*=14 *mm*, the input displacement and output displacement response of the force-dividing block are shown in Figure S2. In this paper, the displacement of the piezoelectric is within 30 *μm*, and the maximum pressure of the piezoelectric is limited. Therefore, in order to improve the force performance of the output terminal, the displacement of the output terminal in the *x* direction is slightly reduced. However, the stiffness analysis shows that the input-output amplification ratio can be adjusted when the *x* and *y* ratios are changed. When *x*<*y*, the device has a displacement amplification function.

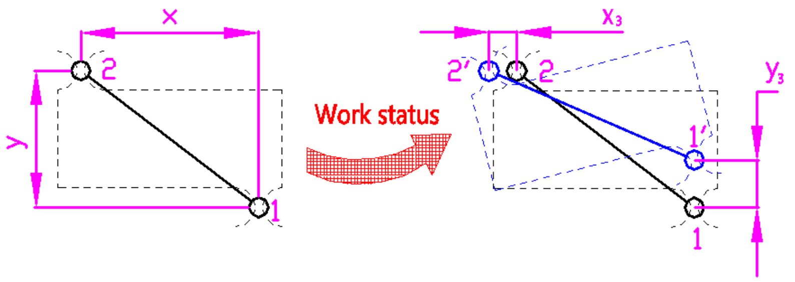

Figure S1. Structure and driving principle of force-dividing block.

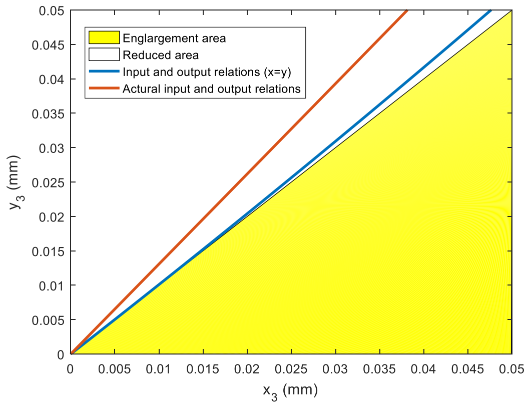

Figure S2. Force-dividing block input-output response.

Rigidity analysis only explains the principle of the relationship between input and output of *x*-direction displacement. In fact, the force-dividing block is a flexible mechanism. In order to predict better the input and output relationship of the device in the *x* direction, in this paper, three sets of stiffness analysis data are selected for amplification, reduction, and critical value for finite element analysis. As shown in Figure S3, a 30 μm displacement constraint for center feed is applied to the end faces of the upper and lower ends of the mechanism, respectively, and a zero displacement constraint is applied to the other five degrees of freedom on the face. Solid model 10 node 187 elements are used in finite element model. The material is an aluminum alloy with elasticity, linearity and isotropy. The elastic modulus is 70 *MPa.* Poisson's ratio is 0.3. Three sets of data selection and FEA results are shown in Table S1. The results show that the amplification ratio increases as the *x*-*y* ratio decreases, but the actual amplification ratio of *x*=*y* is greater than 1.

Table S1. The structure parameters and FEA results.

x (mm)	y (mm)	Input displacement (μm)	Output displacement (μm)	Transmission ratio
18.4	14	30	22.9	0.763
14	14	30	41.8	1.393
12	14	30	52.1	1.737

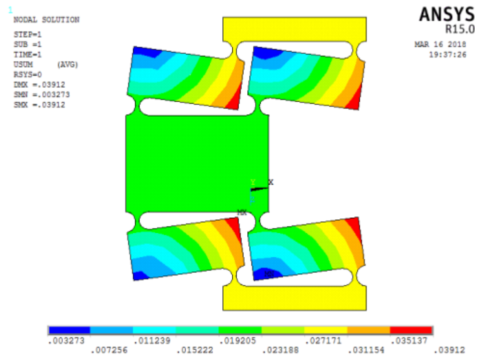

(**a**) *x*=18.4, *y*=14

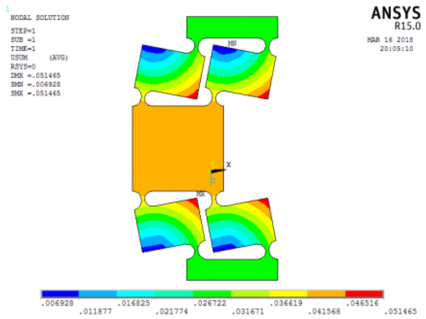

(**b**) *x*=14, *y* =14

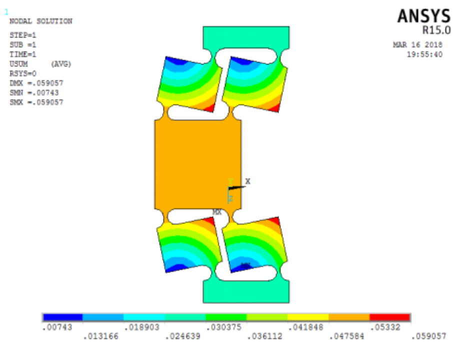

(**c**) *x*=12, *y*=14

Figure S3. The displacement nephogram of FEA.

S2. Error Analytical of Output Terminal

As shown in Figure S4, it is difficult for the output platform center to coincide with the output center of the 2D motion platform. In fact, the deflection error of the output platform mainly comes from the deflection in the *x* and *y* directions at the 2D motion platform output terminal. It is assumed that the center force of the output platform is F. The output platform deflection angle is θ due to the

flexible deformation of the 2D platform. The force is simplified to the midpoint *P* of the 2D motion platform. The equivalent moment of the motion platform midpoint *M* can be expressed as:

$$M = F(\frac{h_1}{2} + L_1 + \frac{h_2}{2}) \tag{S3}$$

$$\theta = \frac{M}{C} \tag{S4}$$

where L_1 is the height distance between the output platform and the 2D motion platform (*mm*), h_1 is the height of the output platform (*mm*), h_2 is the height of the 2D platform (*mm*), and *C* is the rotational stiffness at the output of the 2D motion platform (*Nm/rad*); Assuming that the *z*-direction piezoelectric and output stage are rigid and the 2D motion platform has uniform mechanical properties, the maximum error position at the center point of the output platform varies with L1 as shown in Figure S5. When the height of the output platform and the 2D motion platform are determined, the output platform deflection error becomes smaller with decreasing of L_1.

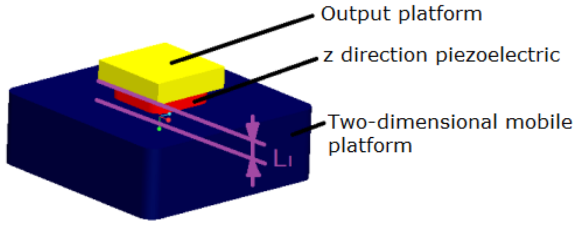

(a) 3D motion device structural solid model

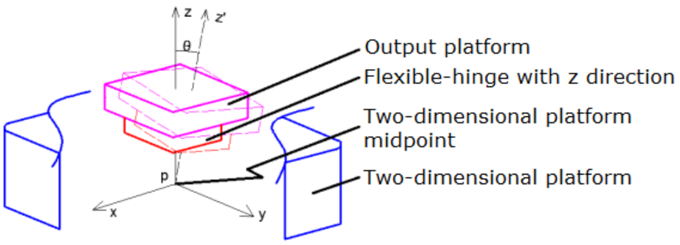

(b) Structural combination model

Figure S4. 3D motion device structure.

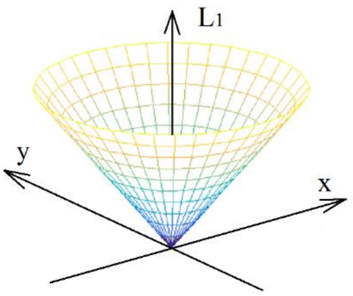

Figure S5. Schematic diagram of output center error position.

S3. Introduction to the MCM Method

As shown in Figure S6, the compliance matrix for a straight round hinge can be expressed as follows:

$$C^R = \begin{bmatrix} \frac{dx}{dF_x} & 0 & 0 & 0 & 0 & 0 \\ 0 & \frac{dy}{dF_y} & 0 & 0 & 0 & \frac{dy}{dM_z} \\ 0 & 0 & \frac{dz}{dF_z} & 0 & \frac{dz}{dM_y} & 0 \\ 0 & 0 & 0 & \frac{d\theta_x}{dM_x} & 0 & 0 \\ 0 & 0 & \frac{d\theta_y}{dF_z} & 0 & \frac{d\theta_y}{dM_y} & 0 \\ 0 & \frac{d\theta_z}{dF_y} & 0 & 0 & 0 & \frac{d\theta_z}{dM_z} \end{bmatrix} \tag{S5}$$

where

$$\frac{dx}{dF_x} = \frac{1}{Eb}\left[\frac{2(2s+1)}{\sqrt{4s+1}}\arctan\sqrt{4s+1} - \frac{\pi}{2}\right],$$

$$\frac{dy}{dF_y} = \frac{12}{Eb}\left[\frac{s(24s^4+24s^3+22s^2+8s+1)}{2(2s+1)(4s+1)^2} + \frac{(2s+1)(24s^4+8s^3-14s^2-8s-1)}{2(4s+1)^{\frac{5}{2}}}\arctan\sqrt{4s+1} + \frac{\pi}{8}\right] + \frac{1}{Gd}\left[\frac{2(2s+1)}{\sqrt{4s+1}}\arctan\sqrt{4s+1}\right],$$

$$\frac{dy}{dM_Z} = -\frac{12}{Eb}\left[\frac{2s^3(6s^2+4s+1)}{(2s+1)(4s+1)^2} + \frac{12s^4(2s+1)}{(4s+1)^{\frac{5}{2}}}\arctan\sqrt{4s+1}\right],$$

$$\frac{dz}{dF_z} = \frac{12R^2}{Ed^3}\left[\frac{2s+1}{2s} + \frac{(2s+1)(4s^2-4s-1)}{2s^2\sqrt{4s+1}}\arctan\sqrt{4s+1} - \frac{2s^2-4s-1)}{8s^2}\pi\right] + \frac{1}{Gd}\left[\frac{2(2s+1)}{\sqrt{4s+1}}\arctan\sqrt{4s+1} - \frac{\pi}{2}\right],$$

$$\frac{dz}{dM_y} = \frac{12R}{Ed^3}\left[\frac{2(2s+1)}{\sqrt{4s+1}}\arctan\sqrt{4s+1} - \frac{\pi}{2}\right],$$

$$\frac{d\theta_y}{dM_y} = \frac{12}{Ed^3}\left[\frac{2(2s+1)}{\sqrt{4s+1}}\arctan\sqrt{4s+1} - \frac{\pi}{2}\right],$$

$$\frac{d\theta_z}{dF_z} = -\frac{12}{EdR}\left[\frac{2s^3(6s^2+4s+1)}{(2s+1)(4s+1)^2} + \frac{12s^4(2s+1)}{(4s+1)^{\frac{5}{2}}}\arctan\sqrt{4s+1}\right],$$

$$\frac{d\theta_z}{dM_z} = \frac{12}{EdR^2}\left[\frac{2s^3(6s^2+4s+1)}{(2s+1)(4s+1)^2} + \frac{12s^4(2s+1)}{(4s+1)^{\frac{5}{2}}}\arctan\sqrt{4s+1}\right],$$

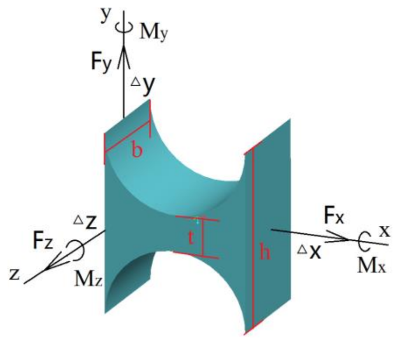

Figure S6. Circular flexure hinge.

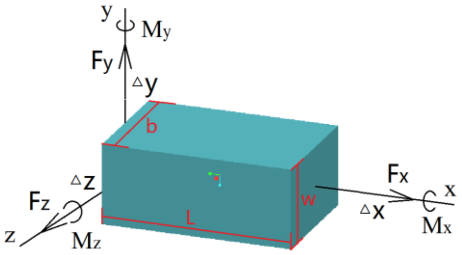

Figure S7. Beam flexure hinge.

As shown in Figure S7, the compliance matrix for straight beam type hinge can be expressed as follows:

$$C^L = \begin{bmatrix} \frac{L}{Ebw^3} & 0 & 0 & 0 & 0 & 0 \\ 0 & \frac{4L^3}{Ebw^3}+\frac{L}{Gbw} & 0 & 0 & 0 & \frac{6L^2}{Ebw^3} \\ 0 & 0 & \frac{4L^3}{Eb^3w}+\frac{L}{Gbw} & 0 & -\frac{6L^2}{Eb^3w} & 0 \\ 0 & 0 & 0 & \frac{L}{Gkbw^3} & 0 & 0 \\ 0 & 0 & -\frac{6L^2}{Eb^3w} & 0 & \frac{12L}{Eb^3w} & 0 \\ 0 & \frac{6L^2}{Ebw^3} & 0 & 0 & 0 & \frac{12L}{Ebw^3} \end{bmatrix} \quad \text{(S6)}$$

where $L(mm)$, $b(mm)$ and $w(mm)$ are the structure parameters, k is a correction coefficient, as shown in Table S2.

Table S2 Correction coefficient k.

b/w	1	2	10	∞
k	0.141	0.229	0.312	0.333

A matrix for the coordinate transform can be written as:

$$T = \begin{bmatrix} R & SR \\ 0 & R \end{bmatrix} \quad \text{(S7)}$$

where the matrix S can be obtained by

$$S = \begin{bmatrix} 0 & -r_z & r_y \\ r_z & 0 & -r_x \\ -r_y & r_x & 0 \end{bmatrix} \quad \text{(S8)}$$

If the matrix R rotates around the z y and x in turn, it is written as:

$$R = \begin{bmatrix} 1 & 0 & 0 \\ 0 & \cos(\alpha) & \sin(\alpha) \\ 0 & -\sin(\alpha) & \cos(\alpha) \end{bmatrix} \bullet \begin{bmatrix} \cos(\beta) & 0 & -\sin(\beta) \\ 0 & 1 & 0 \\ \sin(\beta) & 0 & \cos(\beta) \end{bmatrix} \bullet \begin{bmatrix} \cos(\varepsilon) & \sin(\varepsilon) & 0 \\ -\sin(\varepsilon) & \cos(\varepsilon) & 0 \\ 0 & 0 & 1 \end{bmatrix} \quad \text{(S9)}$$

where α, β and ε are rotation angles rounding axis x y and z respectively.
